# 7-Ketocholesterol-Induced Inflammation Signals Mostly through the TLR4 Receptor Both *In Vitro* and *In Vivo*

**DOI:** 10.1371/journal.pone.0100985

**Published:** 2014-07-18

**Authors:** Jiahn-Dar Huang, Juan Amaral, Jung Wha Lee, Ignacio R. Rodriguez

**Affiliations:** Mechanisms of Retinal Diseases Section, Laboratory of Retinal Cell and Molecular Biology, National Eye Institute, National Institutes of Health, Bethesda, Maryland, United States of America; Massachusetts General Hospital and Harvard Medical School, United States of America

## Abstract

The cholesterol oxide 7-ketocholesterol (7KCh) has been implicated in numerous age-related diseases such as atherosclerosis, Alzheimer's disease, Parkinson's disease, cancer and age-related macular degeneration. It is formed by the autooxidation of cholesterol and especially cholesterol-fatty acid esters found in lipoprotein deposits. This molecule causes complex and potent inflammatory responses *in vitro* and *in vivo*. It is suspected of causing chronic inflammation in tissues exposed to oxidized lipoprotein deposits. In this study we have examined the inflammatory pathways activated by 7KCh both in cultured ARPE19 cells and *in vivo* using 7KCh-containing implants inserted into the anterior chamber of the rat eye. Our results indicate that 7KCh-induced inflammation is mediated mostly though the TLR4 receptor with some cross-activation of EGFR-related pathways. The majority of the cytokine inductions seem to signal via the TRIF/TRAM side of the TLR4 receptor. The MyD88/TIRAP side only significantly effects IL-1β inductions. The 7KCh-induced inflammation also seems to involve a robust ER stress response. However, this response does not seem to involve a calcium efflux-mediated UPR. Instead the ER stress response seems to be mediated by yet identified kinases activated through the TLR4 receptor. Some of the kinases identified are the RSKs which seem to mediate the cytokine inductions and the cell death pathway but do not seem to be involved in the ER stress response.

## Introduction

7-Ketocholesterol (7KCh) is a naturally occurring cholesterol oxide formed by the autooxidation of cholesterol (Ch) and cholesterol-fatty acid esters [1]. It is commonly found in oxidized lipoprotein deposits associated with atheromatous plaques [2]–[4] as well as in lipoprotein deposits in Bruch's membrane and choriocapillaris in the back of the retina [5]. It has been shown to be the major cytotoxic component in oxidized LDL [6]. This oxysterol is known to be highly inflammatory both *in vitro* [7], [8] and *in vivo* [9]. Its inflammatory and cytotoxic properties have been implicated in the pathogenesis of numerous aging diseases [8], including atherosclerosis [4], [7], Alzheimer's disease [8], [10], cancer [11], Parkinson's disease [10] and age-related macular degeneration [10], [12].

7-KCh has been shown to activate numerous inflammatory pathways [13], [14]. This seems to depend on the particular *in vitro* model being investigated. It can induce endoplasmic reticulum (ER) stress [15], stimulation of Akt [16], cell proliferation through the epidermal growth factor receptor (EGFR) [17] and activation of the Toll-like receptor 4 (TLR4) [18], to mention a few. The consensus in the published literature is that NFκB- mediated cytokine production is the main pathway responding to 7KCh-induced inflammation.

In this study we have examined most of the major inflammatory pathways suspected of being activated by 7KCh. Our data indicates that while several downstream pathways may be involved in the inflammatory signaling, the majority of the inflammation occurs via TLR4 pathway both *in vitro* and *in vivo*.

## Materials and Methods

### Materials

Ch and 7KCh were purchased from Steraloids Inc. (Newport, RI). LY294002, wortmannin, SB203580, U0126, SP600125, Ac-YVAD-CMK, AG1478, TBB, SL0101 were purchased from EMD Millipore (Billerica, MA). Amlexanox was from Tocris Bioscience (Bristol, UK). ST2825 was from MedChem Express (Monmouth Junction, NJ). BI-D1870 was from Enzo Life Sciences, Inc. (Farmingdale, NY). CLI-095 was from Invivogen Inc. (San Diego, CA). Lipopolysaccharide (LPS) from Salmonella, IRAK inhibitor1/4 and necrostatin-1were purchased from Sigma-Aldrich (St. Louis, MO). Sterculic acid was purchased from Biofine (Vancouver, Canada).

### Definition and quantification of 7KCh-induced inflammation

In this study 7KCh-induced inflammation is defined by the induction of six inflammatory markers, four cytokines, IL-1β, IL-6, IL-8, VEGF, the transcription factor CHOP (CCAAT-enhancer-binding protein homologous protein and the molecular chaperone GRP78 (78 kDa glucose-regulated proteins). The latter two are known as ER-stress markers. The mRNA induction was measured by quantitative real-time PCR (qRT-PCR) Protein responses were determined by immunoblot for CHOP and GRP78. Cytokines protein responses were determined using the Luminex XMAP technology and a MAGPIX instrument (Luminex Corp, Austin, TX).

### Cell cultures and preparation of 7KCh

ARPE19 cells are an immortalized human retinal pigment epithelium (RPE) cell line obtained from American-Type Culture Collection (Manassas, VA). They were grown in 24-well plates in complete medium (DMEM/F-12 medium (Mediatech, Tewksbury, MA) containing 10% FBS, 100 IU/ml penicillin, and 100 µg/ml streptomycin (Life Technologies, Grand Island, NY). Approximately 1×10^5^ cells were seeded in each well for 16-24 hr until the cell confluence reached approximately 90–95%. Each test point was performed in quadruplicates (6 measurements per plate) which were then pooled. Each experiment repeated three or more times.

Stock solutions of 7KCh and Ch (10 mM) were prepared in 45% hydroxypropyl-β-cyclodextrin (Sigma-Aldrich, St. Louis, MO) as previously described [5]. Working solutions (1 mM) were prepared by diluting the stock solutions 1 to 10 in 1x PBS. The 7KCh and Ch must be added directly to the well containing the cells. Pre-dilution in media will results in loss of the sterols to the plastic container walls resulting in loss of toxicity for 7KCh and great variation in results.

### Testing of 7KCh antagonists

Once the desired cell confluence was reached (90–95%), the cells were incubated in serum-free medium with the various inhibitors for 1 hr. 7KCh (8 µM) was then added to the cell culture and continued the incubation for 24 hr. Cells were then collected and RNA prepared for qRT-PCR.

In cases where secreted cytokines were measured, the cells were treated with 6 µM 7KCh for 48 hr as it required longer treatment time to see the secreted proteins. At the end of 7KCh treatment, conditioned medium was collected, centrifuged at 1,000 rpm to remove cell debris, then frozen and stored for the quantification of cytokine levels. Cells lysates were also prepared for immunoblot analyses.

### Gene knockdown with siRNA transfection and 7KCh treatment

The siRNAs (CHOP, s3997-4392420; ATF4, s1703; PI3K subunit P110α, s10520; β-catenin, s436; NRLP3, s41554) were purchased from Ambion (Life Technologies, Grand Island, NY). Cells were transfected using the Amaxa cell transfection kit (Lonza, Colonge, Germany). The cells were allowed to recover in complete medium for approximately 16–24 hr in order to reach 90–95% cell confluency. The transfected cells were then treated with 7KCh in serum-free medium for 24 hr. Cells were then lysed and pooled for mRNA and/or protein expression.

### Gene overexpression with adenovirus transfection and 7KCh treatment

Approximately 0.5×10^5^ ARPE19 cells were seeded and grown in the complete medium (24-well plate) for 20 hr. The cells were then incubated with adenovirus (MOI  = 20) in serum-free medium for 48 hr in order to overexpress dominant negative IκBα (dnIκBα), mitogen-activated protein kinase phosphatase 2 (MKP2), Toll interacting protein (TOLLIP), tumor necrosis factor receptor associated factor (TRAF1), suppressor of cytokine signaling SOCS1, SOCS2 and SOCS3(Vector Biolabs, Philadelphia, PA). Adenovirus that expresses enhanced GFP (Vector Biolabs) was used as control. The transfected cells were then treated with 7KCh for 24 hr. Conditioned medium was collected, centrifuged at 1,000 rpm to remove cell debris, then frozen and stored for the quantification of secreted cytokine levels. Cells were lysed and pooled for the examination of mRNA or protein expression. We did not detect any cell death due to adenovirus transfection at the end of the experiment.

### Quantitative real-time PCR

The RNA was extracted using RNeasy Mini Kit (Qiagen, Valencia, CA). The reverse transcription was performed with reagents and kits from Life Technologies (Grand Island, NY). Quantification of mRNA expression was performed using the Taqman gene expression assays purchased from Applied Biosystems, Inc. (Carlsbad, CA) with the following primers (VEGFa Hs00173626_m1, IL-1β Hs01555413_m1, IL-6 Hs00174131_m1, IL-8 Hs00174103_m1, CHOP Hs00358796_g1, GRP78 Hs00607129_gH, PERK Hs00984006_m1, IRE1 Hs00176385_m1, ATF4 Hs00909569_g1, XBP-1 Hs00231936_m1, EIF2a Hs00230684_m1, P58IPK Hs00534483_m1, SOCS1 Hs00705164_s1, SOCS2 Hs00919620_m1, SOCS3 Hs02330328_s1, GAPD 4352934e).

GAPD expression was used as an endogenous standard. All qRT-PCR experiments were measured in an ABI 7900 Real-Time PCR Instrument (Applied Biosystems).

### Immunoblots

The ARPE19 cell lysate was prepared by using MPER buffer solution (Thermo Fisher, Waltham, MA) containing cOmplete Protease Inhibitor Cocktail (1 tablet per 50 ml) (Roche Applied Science). The proteins were separated by SDS-PAGE on 10% Bis-Tris gels (Invitrogen). The gels were blotted onto nitrocellulose membranes (Invitrogen). The blots were probed with primary antibodies to P110α, phospho-Akt, Akt, phospho-P38 MAPK, phospho-ERK1/2, phospho-JNK, CHOP, GRP78 (all 1∶1000, Cell Signaling, Danvers, MA), or GAPDH (1∶2000, Abcam, Cambridge, MA) at 4°C overnight. The membranes were then further incubated with anti-rabbit IgG, HRP-linked antibodies (1∶2000, Cell Signaling) at room temperature for 1 h. The membranes were then developed in Chemiluminescent Substrate (Thermo Fisher) and the proteins were visualized on Kodak X-ray films (Carestream Health, Rochester, NY).

### Secreted cytokine assay

The level of secreted VEGF, IL-1β, IL-6, and IL-8 in the conditioned medium was quantified using MILLIPLEX MAP Human Cytokine/Chemokine Panels (Millipore) in a MAGPIX system (Luminex, Austin, TX) according to the manufacturer's protocol. Briefly, 25 µl of each sample was incubated with a mixture of magnetic beads coated with antibodies specific to each target cytokine/chemokine in a 96-well panel at 4°C overnight. The beads mixture with attached cytokine was washed twice and then incubated with detection antibody solution for 1 hr at room temperature. We then added 25 µl of the streptavidin-phycoerythrin solution to the panel and incubated for 30 min at room temperature. The beads were washed twice and re-suspended in the drive fluid and the panel was transferred into the MAGPIX instrument. The signal intensity of each cytokine/chemokine was then determined based on a parallel standard curve created in the panel using Luminex xMAP software (Luminex) and analyzed with MILLIPLEX Analysis software version 3 (Millipore).

### Cell viability assay

Cell viability was determined by the dehydrogenase activity of the ARPE19 cells using Cell Counting Kit-8 (Dojindo, Rockville, MD) according to manufacturer's protocol.

### Kinome*scan* kinase competitive inhibition assay

This is a proprietary fee for service competitive inhibition assay performed by DiscoverX (www.discoverx.com). For details go to http://www.discoverx.com/technologies-platforms/competitive-binding-technology/kinomescan-technology-platform.

### *In vivo* angiogenesis assay

The *in vivo* angiogenesis assay was performed as previously described [9]. In brief, wafers were made containing a mixture of 7% 7KCh (w/w), containing various test compounds (usually 5–12% w/w) and the remaining an equal mixture of polyethylene glycol (MW: 20,000) and hydrogel (2-hydroxyethylmethacrylate). A small amount of phenol red (0.1%) is added to visualize and ensure complete mixing. The mixtures were dissolved in ethanol then slowly dried in a nitrogen stream until a paste forms. The paste is thoroughly mixed then flashed dried under vacuum using a lyophilizer. The dried powder is then weighed and pressed by 22 tons of pressure using a hydraulic press (Specac, Sweedesboro, NJ). Implants are made using a trephine (0.5 mm, id).

A corneal incision is made in rat eyes and the implants placed on top of the iris. In implants containing 7% 7KCh only, angiogenesis begins at day 4 and peaks between days 7–10, then it begins to wanes. The angiogenesis is quantified using images of the fluorescein angiography and the vessels area (in mm^2^) is quantified using software as previously described [9].

The animal study protocol to insert 7KCh-implants into the rat anterior chamber was approved by the National Eye Institute's Animal Care and Use Committee in accordance with the National Institutes of Health guidelines for Animal Care and Use. All implantation was performed under anesthesia as previously described [9].

### Statistics

Statistical comparisons between groups were performed using two-tailed Student's *t*-test. We consider the result as significant when *p*<0.05.

## Results

### 7KCh-induced inflammation is dependent on intercellular phosphorylation of kinases

Treatment of ARPE19 cells with 7KCh increases the levels of protein-bound phosphate by 10% (data not shown). Sterculic acid has been previously reported to antagonize 7KCh-induced inflammation and cell death [19]. Simultaneous treatment of ARPE19 cells with 7KCh (10 µM) and sterculic acid (1 µM) prevents the 7KCh-induced intercellular protein phosphorylation (data not shown).

**Figure 1:**
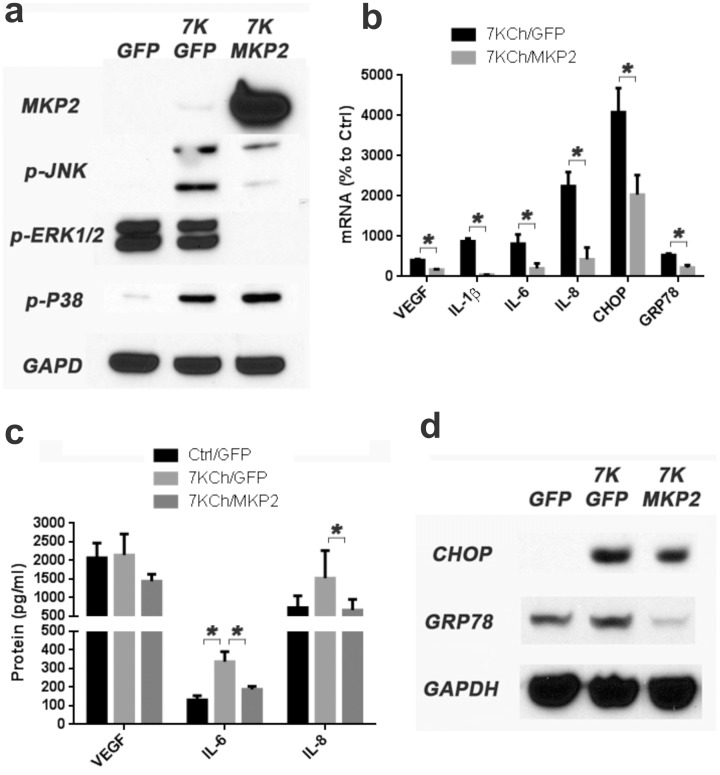
Effect of MKP2 overexpression on 7KCh-mediated inflammation. Overexpression was achieved by transducing the ARPE19 cells with a commercially available adenovirus expressing MPK2. ARPE19 cells were treated with 8 µM 7KCh for 24 hr and the mRNA inductions of the inflammatory markers were measured by qRT-PCR. **(a)** Immunoblots demonstrating the overexpression of MKP2 and the effects on the phosphorylation of JNK and ERK1/2 and P38 MAPK**.** JNK and p38 were induced by 7KCh treatment but there was no significant increase of phosphorylated ERK1/2 at the 24 hr time point. MKP2 significantly reduced the formation p-JNK and p-ERK1/2 but had no effect on p38. **(b)** QRT-PCR of the inflammatory markers (mean ± s.d., *n*≥3) with and without the overexpression of MKP2. The overexpression of MKP2 suppressed the induction of IL-1β (8.8 to 0.4 fold), IL-6 (8.1 to 2.0 fold), IL-8 (22.5 to 4.3 fold), VEGF (4.1 to 1.7 fold), CHOP (40.9 to 20.4 fold), and GRP78 (5.4 to 2.2 fold). **(c)** The secreted cytokine levels were measured using the Luminex XMAP technology in the conditioned medium after treatment with 6 µM 7KCh for 48 hr (VEGF, *n* = 3) or 8 µM 7KCh for 24 hr (IL-6 and IL-8, *n* = 4) with and without MKP2 overexpression (mean ± s.d.). MKP2 overexpression reduced the secreted cytokine level for VEGF (2145 pg/ml to 1442 pg/ml), IL-6 (337 pg/ml to 188 pg/ml) and IL-8 (1523 pg/ml to 662 pg/ml). **(d)** MKP2 overexpression reduced the induction of CHOP and GRP78. GFP overexpression was used as control. *p<0.05, two-tailed Student's t-test.

To further demonstrate the phosphorylation effect MAPK phosphatase 2 (MKP2) was overexpressed in ARPE19 cells by transducing with a replication negative adenovirus containing the MKP2 gene (Fig. 1). MKP2 is known to dephosphorylate various activated kinases downstream of multiple inflammatory pathways [20], [21] and thus attenuating the inflammatory response.

The immunoblot (Fig. 1a) demonstrated a robust overexpression of MKP2. This overexpression significantly reduced the 7KCh-induced p-JNK levels, ablated p-ERK1/2 but had no effect on p-P38 (Fig. 1a). Interestingly, treatment with 7KCh alone caused a significant induction of p-JNK and p-p38 but had no effect on ERK1/2 (Fig. 1a). Overexpression of MKP2 had a very significant effect at attenuating the mRNA induction of all of the inflammatory markers (Fig. 1b). Similar effects were observed for the secreted cytokines in the conditioned media (Fig. 1c). Immunoblots also demonstrate significant reduction in CHOP and GRP78 (Fig. 1d). This further demonstrates that 7KCh-induced inflammation is dependent on intracellular phosphorylation of kinases.

### Mitogen activated kinases (MAPKs) are not involved in mediating 7KCh-induced inflammation

**Figure 2:**
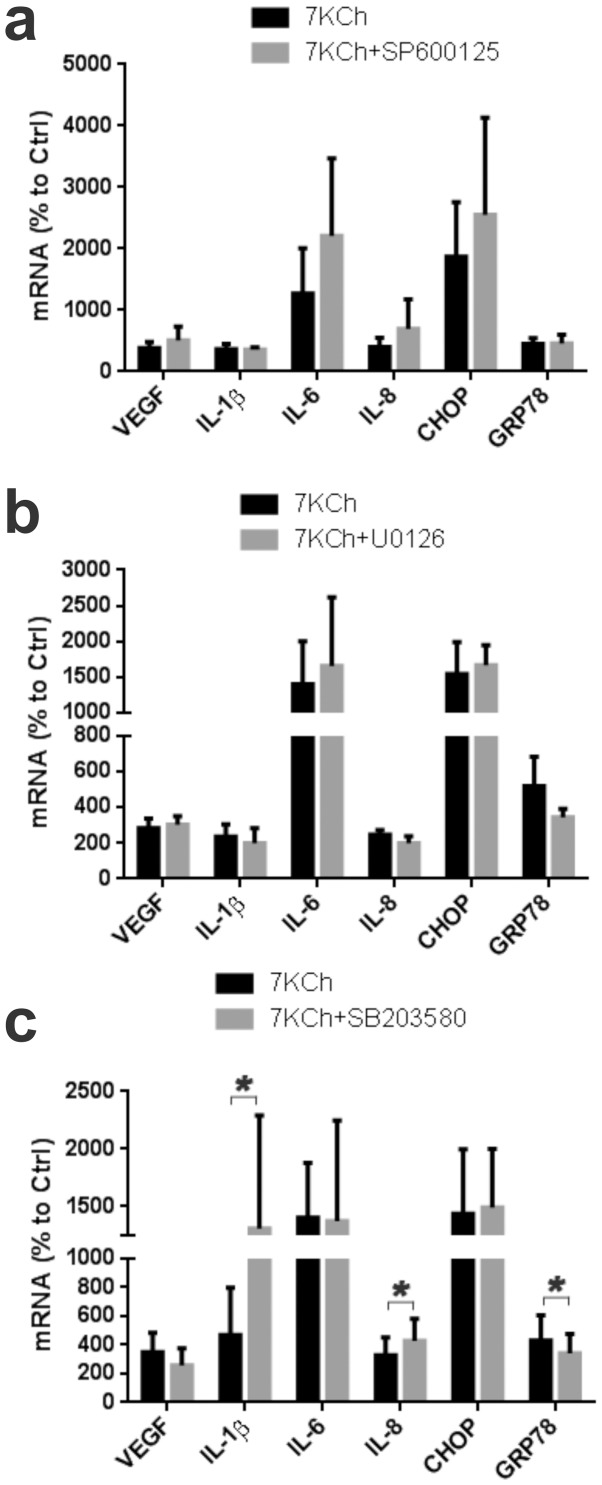
Effect of MAPK inhibition on 7KCh-induced inflammation. ARPE19 cells were treated with 8 µM 7KCh for 24 hr and the mRNA inductions of the inflammatory markers were measured by qRT-PCR**. (a)** Measurements (mean ± s.d., *n* = 3) with and without 5 µM SP600125 (a JNK inhibitor). SP600125 did not suppress any of the inflammatory markers but rather enhanced the induction of IL-6 (12.7 to 22.1 fold) and CHOP (18.7 to 25.5 fold). **(b)** Measurements (mean ± s.d., *n*≥3) with and without the 10 µM U0126 (ER1/2 inhibitor). U0126 did not suppress any of the 7KCh-induced inflammatory markers. **(c)** Measurements (mean ± s.d., *n*≥5) with and without 10 µM SB203580 (p38MAPK inhibitor). SB203580 slightly reduced 7KCh-induced VEGF expression (3.5 to 2.6 fold) but increased the expression of IL-1β (4.7 to 13.1 fold) and IL-8 (3.3 to 4.3 fold). SB203580 had no effect on CHOP induction but demonstrated a slight but statistically inhibition of the GRP78 induction (4.3 to 3.4 fold). *p<0.05, two-tailed Student's t-test.

To further define the effect of MKP2 we used specific inhibitors of various mitogen activated protein kinases (MAPKs) known to be substrates for MKP2 (cJun, MEK1/2 and to a lesser extent p38MAPK) (22). MAPKs are a large family of enzymes that mediate a wide variety of inflammatory responses [22]. Previously published work from our group [14] and others [10], [13], [14] have also implicated various MAPK pathways in the 7KCh-induced inflammatory responses. The cJun inhibitor SP600125 [23] had no effect on VEGF, IL-β or GRP78 mRNA expression although there may be an increased response from IL-6 and CHOP (not statistically significant) (Fig. 2a). The MEK1/2 inhibitor U0126 [24] had no significant effect on any of the inflammatory markers (Fig. 2b). The p38MAPK inhibitor SB203580 [25] also had no significant effect on VEGF, IL-6 or GRP78 but showed statistically significant increase in IL-1β from 4.1 to 13.1-fold and IL-8 from 3.3 to 4.3-fold. It also showed a statistically significant decrease in GRP78 from 4.3 to 3.3-fold (Fig. 2c). The inhibition of cJun and p38MAPK demonstrate a slight potentiating effect on some of the inflammatory markers suggesting they may be involved in downregulating some of the NFκB transcriptional responses.

### Inhibition of NFκB activity suppresses 7KCh-induced inflammation

**Figure 3:**
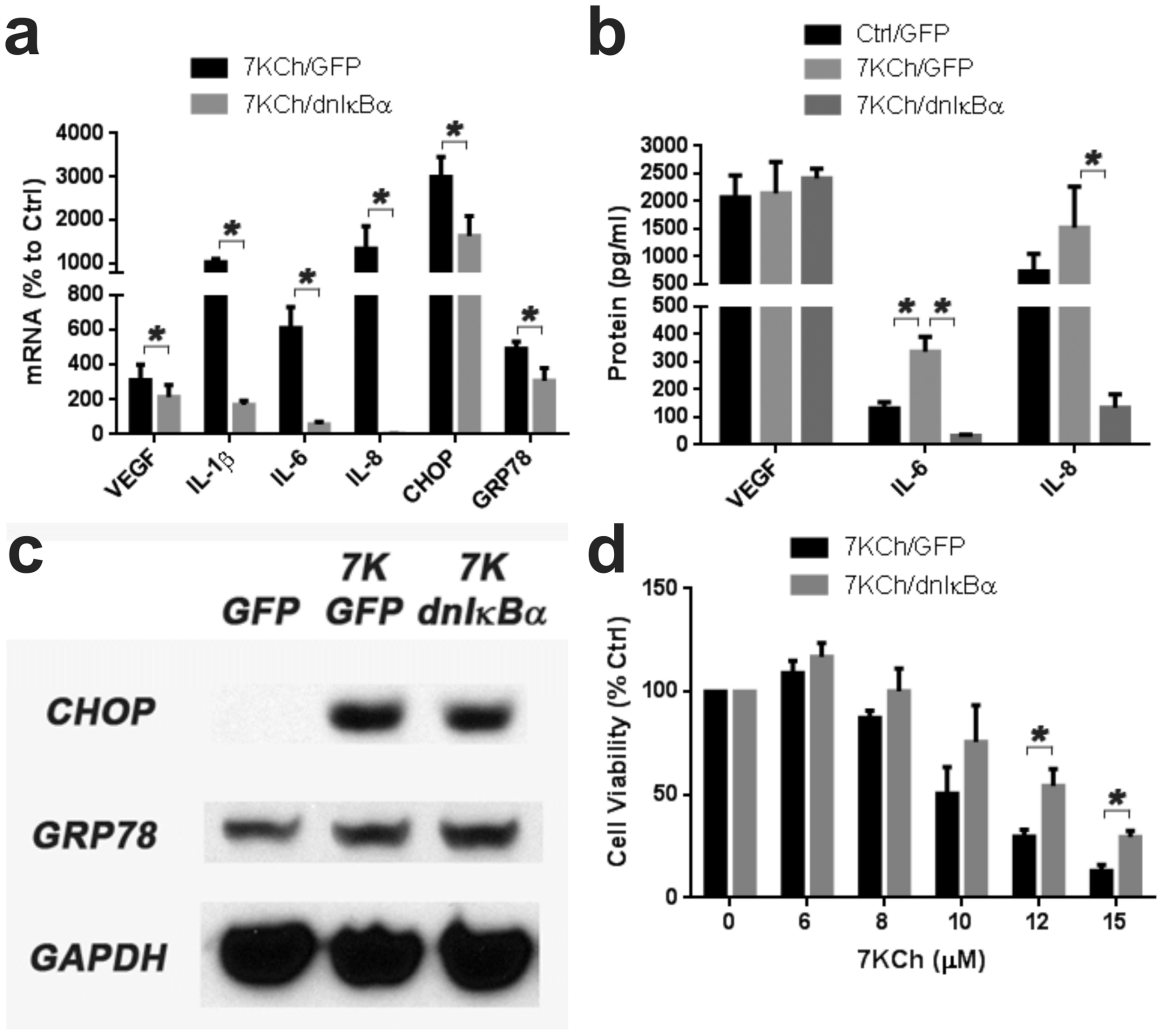
Effect of dnIκBα overexpression on 7KCh-induced inflammation and cell death. ARPE19 cells were transduced with a commercially available adenovirus expressing a dominant negative IκBα (dnIκBα). After transduction cells were treated with 8 µM 7KCh for 24 hr and the mRNA inductions of the inflammation markers measured by qRT-PCR**. (a)** Measurements (mean ± s.d., *n* = 5) of the inflammatory markers with and without dnIκBα overexpression. The dnIκBα overexpression reduced the induction of VEGF (3.1 to 2.2 fold), I-1β (10.3 to 1.7 fold), IL-6 (6.1 to 0.6 fold), IL-8 (13.5 to 0.02 fold), CHOP (30.0 to 16.5 fold) and GRP78 (4.9 to 3.1 fold). **(b)** Measurement of the secreted cytokines in the conditioned medium after treatment with 6 µM 7KCh for 48hr (VEGF, *n* = 3) or 8 µM 7KCh for 24 hr (IL-6 and IL-8, *n* = 4) with and without dnIκBα overexpression (mean ± s.d.). The overexpression of dnIκBα suppressed the 7KCh-induced secretion of both IL-6 (337 pg/ml to 33 pg/ml) and IL-8 (1523 pg/ml to 133 pg/ml). **(c)** Immunoblot demonstrating the expression of CHOP and GRP78 with and without overexpression of dnIκBα. A slightly reduction in the induction of CHOP was observed but there was no effect on GRP78. **(d)** Cell viability measurements (mean ± s.d., *n* = 3) in response to 6-15 µM 7KCh with and without dnIκBα overexpression. Overexpression of dnIκBα protected the cells from 7KCh-induced cell death. The overexpression of GFP was used as control. *p<0.05, two-tailed Student's t-test.

NFκB is a high level transcription factor known to be essential in mediating cytokine expression and that of other inflammatory markers [26]. Using a replication negative adenovirus coding for a dominant negative IκBα (dnIκBα) ARPE19 cells were transduced as previously described [27]. The overexpression of dnIκBα essentially ablated the NFκB activity which in turn ablated the mRNA expression of IL-1β, IL-6 and IL-8 (Fig. 3a). However, the effect on the mRNA expression of VEGF and the ER stress markers CHOP and GRP78 was less significant (Fig. 3a). At the protein level, the expression of IL-6 and IL-8 were also markedly reduced but VEGF did not change (Fig. 3b). No significant change was observed in the protein levels of CHOP and GRP78 (Fig. 3c). Inhibition of NFκB provided significant protection from 7KCh-induced cell death (Fig.3d). This demonstrates that most of the 7KCh-induced cytokine expression is mediated by NFκB. It also suggests that VEGF, CHOP and GRP78 mRNA expression is only partially dependent on NFκB and may involve other transcription factors. The partial protection from cell death suggests that the 7KCh-induced cell death pathway is influenced but not fully controlled by NFκB activation.

### Phosphoinositides 3-kinases (PI3Ks) and Akt are not involved in 7KCh-induced inflammation

**Figure 4:**
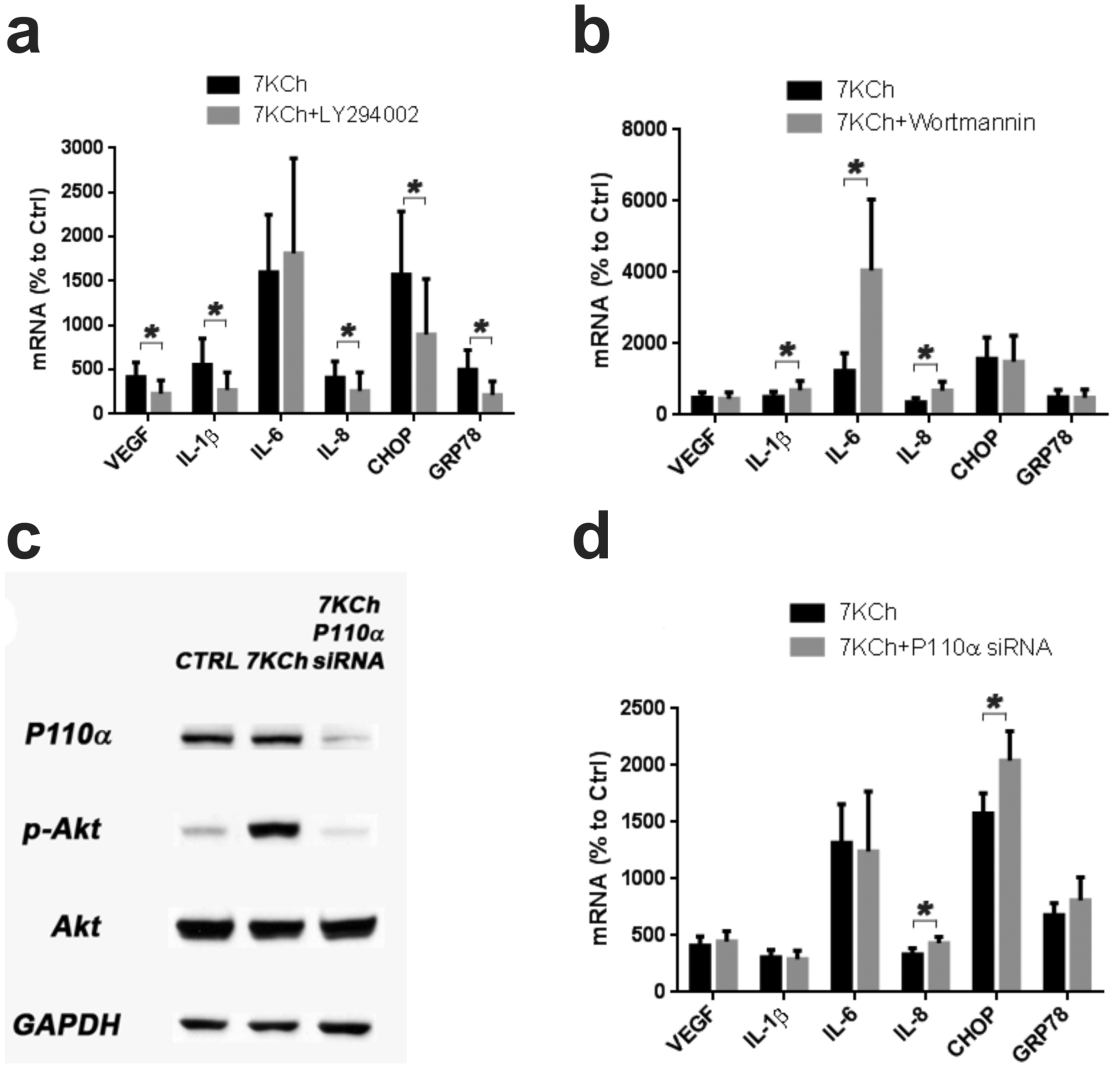
7KCh-induced inflammation is independent of PI3K-Akt activation. ARPE19 cells were treated with 8 µM 7KCh for 24 hr and the mRNA inductions of the inflammatory markers were measured by qRT-PCR **(a)** Measurements (mean ± s.d., *n*≥9) with and without 10 µM LY294002. LY294002 significantly suppressed the mRNA induction of five of the six inflammatory markers (VEGF: 4.2 to 2.3 fold, IL-1β: 5.6 to 2.7 fold, IL-8: 4.1 to 2.6 fold, CHOP: 15.7 to 9.0 fold, and GRP78: 5.0 to 2.2 fold) but had little effect on the the IL-6 induction (16.0 to 18.1 fold). **(b)** Measurements (mean ± s.d., *n* = 5) with and without 1 µM Wortmannin. Wortmannin did not inhibit the induction of the inflammatory markers, instead it increased the mRNA induction of IL-1β (5.0 to 6.9 fold), IL-6 (12.3 to 40.6 fold), and IL-8 (3.5 to 6.8 fold). It had no effect on VEGF, CHOP or GRP78. **(c)** Immunoblot demonstrating the effect of the knockdown of P110α (catalytic subunit of PI3K) with a siRNA. The P110α knockdown inhibited the phosphorylation of Akt induced by 6 hr of 8 µM 7KCh treatment. The 6 hr time point was determined in prior experiments as the peak in 7KCh-induced Akt phosphorylation. A scrambled siRNA was used as control. **(d)** Measurements (qRT-PCR) (mean ± s.d., *n* = 3) of the inflammatory markers with and without P110α knockdown. P110α knockdown did not change the mRNA induction of VEGF, IL-1β, IL-6, and enhanced the induction of IL-8 (3.3 to 4.3 fold), CHOP (15.7 to 20.4 fold), and GRP78 (6.8 to 8.1 fold). *p<0.05, two-tailed Student's t-test.

In a previous study we reported that phosphatidylinositol 3-kinase (PI3K) was involved in 7KCh-induced inflammation [14]. This was based on results obtained with the PI3K inhibitor LY294002 [28]. LY294002 was found to inhibit 7KCh-mediated cytokine induction and ER stress [14], [19]. These results were reproducible and LY294002 attenuated the 7KCh-induced mRNA response for VEGF, IL-1β, IL-8, CHOP and GRP78, but had no effect on IL-6 (Fig. 4a). However, another PI3K inhibitor Wortmannin [29] had no effect and demonstrated a statistically significant increase in the induction of IL-1β, IL-6 and IL-8 (Fig. 4b). This inhibitor is highly specific for PI3K and is effective at considerably lower concentrations [29]. This prompted us to further investigate the involvement of the PI3K-Akt pathway. A siRNA was used to nearly ablate the protein expression of P110α (the catalytic subunit of PI3K) which in turn also ablated the phosphorylation of Akt (Fig. 4c). The knockdown of P110α had no effect on the mRNA expression of the cytokines and GRP78 (Fig. 4d). However, it did cause a slight but statistically significant increase in IL-8 and CHOP (Fig. 4d). Except for the IL-6 increase, this was similar to the results obtained with Wortmannin (Fig. 4b). Thus, the inhibition of PI3K activity had no significant effect on antagonizing 7KCh-induced inflammation.

**Figure 5:**
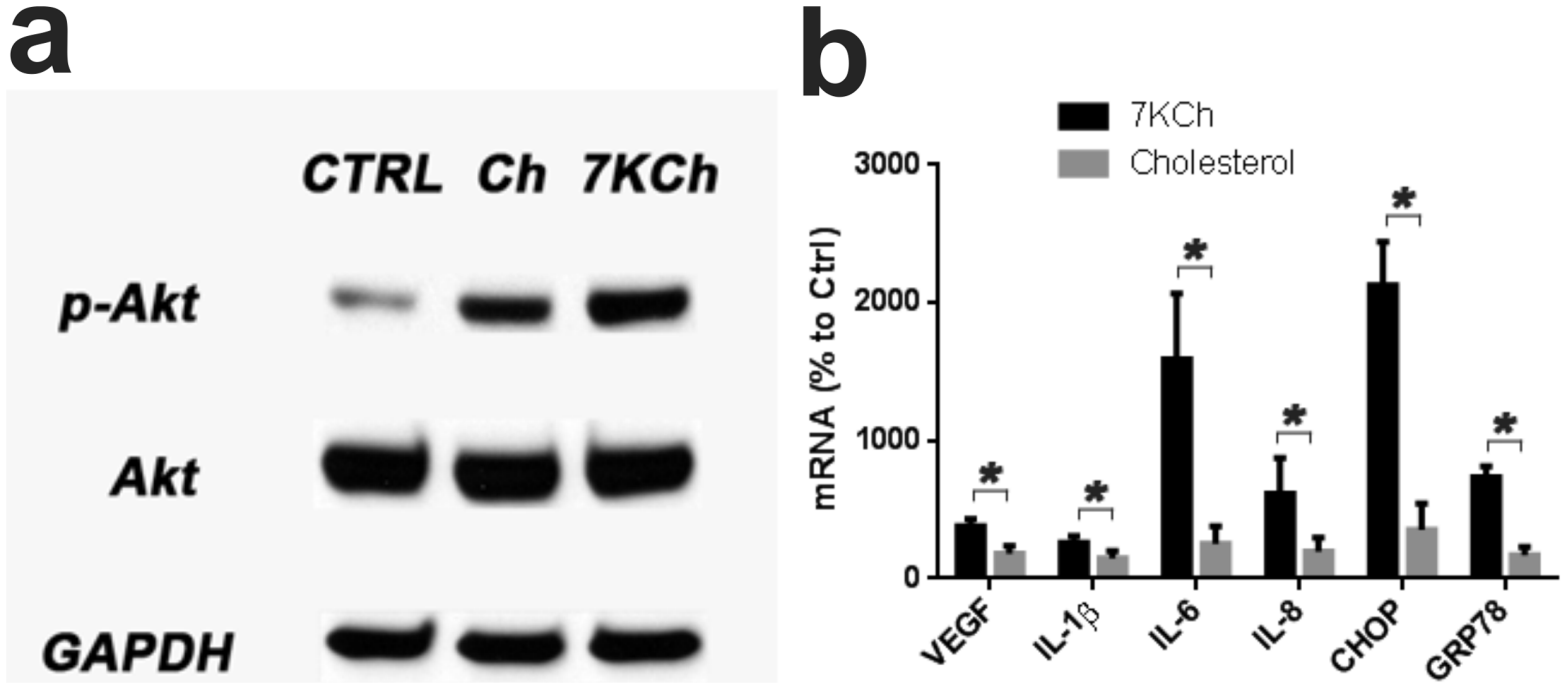
Cholesterol induces PI3K-Akt activation with no inflammatory response. ARPE19 cells were treated with 8 µM Ch or 7KCh for 6 hr. **(a)** Immunoblot demonstrating significant phosphorylation of Akt by both treatments. **(b)** qRT-PCR measurements of the inflammatory markers (mean ± s.d., *n* = 3) after treatment with 8 µM Ch and 7KCh for 24 hr. Cholesterol causes significant phopsphorylation of Akt but no significant inflammatory response. This is additional evidence indicating that PI3K-Akt signaling is not involved in the 7KCh-induced inflammatory response. *p<0.05, two-tailed Student's t-test.

Akt is a very important kinase that works downstream of PI3K and upstream of NFκB and is known to be involved in multiple signaling pathways [30]. Since there is considerable “cross-talk” between inflammatory pathways we wanted to further confirm that Akt was not involved in mediating 7KCh-induced inflammation. To demonstrate this we treated ARPE19 cells with Ch and 7KCh which both cause a significant phosphorylation of Akt (p-Akt) (Fig. 5a). However, unlike 7KCh, Ch causes no inflammatory responses when given to cells (Fig. 5b). We have also previously shown that Ch causes no inflammation or angiogenesis *in vivo* using our anterior chamber rat model (9). This further demonstrates that the phosphorylation/activation of Akt has no direct effect on 7KCh-induced inflammatory responses.

### Other potential pathways inhibited by LY294002

Since LY294002 proved to be a potent antagonist to 7KCh-induced inflammation (Fig. 4a), we examined other potential pathways previously shown to be inhibited by LY294002 [31]. Protein kinase 2 (also known as casein kinase 2, or CK2) has been reported to be inhibited by LY294002 [32]. LY294002 has also been shown to suppress the expression of β-catenin and multiple components of the Wnt/β-catenin pathways [33].

**Figure 6:**
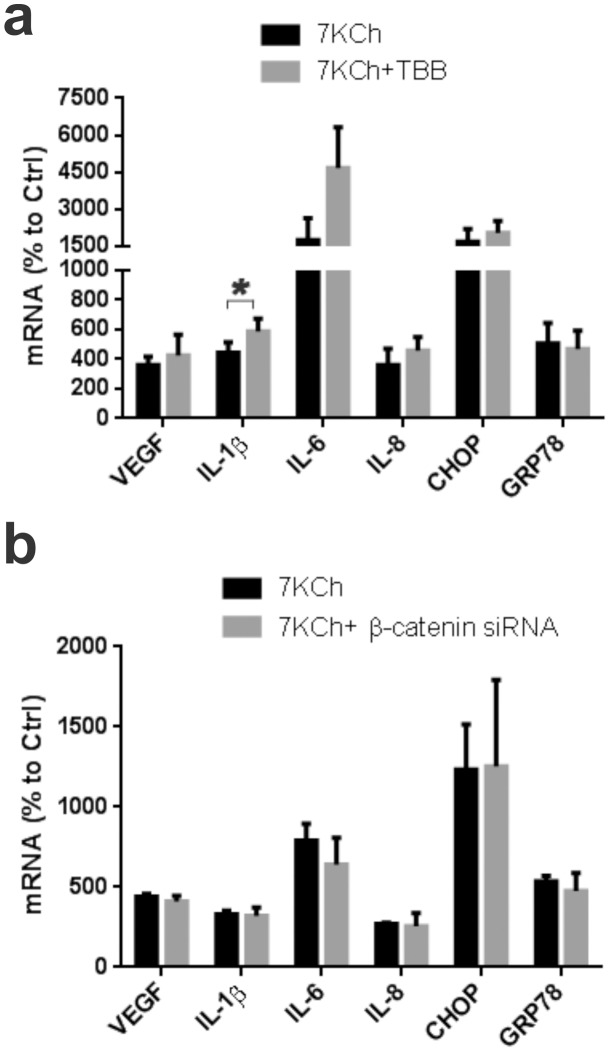
Protein kinase CK2 and β-catenin do not mediate 7KCh-induced inflammation. ARPE19 cells were treated with 8 µM 7KCh for 24 hr and the mRNA inductions of the inflammatory markers were measured by qRT-PCR. **(a)** Measurements (mean ± s.d., *n* = 3) with and without 5 µM TBB (CK2 inhibitor). TBB treatment caused a slight increase in IL-1β (4.4 fold to 5.9 fold) and a significant increase in IL-6 induction (17.5 fold to 46.6 fold). **(b)** Measurements (mean ± s.d., *n* = 3) with and without siRNA knockdown of β-catenin (Wnt signaling). Knockdown of β-catenin had no effect on 7KCh-induced inflammation. *p<0.05, two-tailed Student's t-test.

Protein kinsase CK2 is a highly pleiotropic Ser/Thr specific protein kinase with a very wide variety of substrates [34]. Inhibition of CK2 with the specific inhibitor 4, 5, 6, 7-tetrabromobenzotriazole (TBB) [33] did not suppress the 7KCh-induced inflammation (Fig. 6a). On the contrary, it increased IL-1β mRNA expression from 4.4 to 5.9-fold and IL-6 from 17.5 to 46.6-fold. Therefore, the antagonizing effect of LY294002 against 7KCh-induced inflammation is not through the inhibition of CK2

The protein β-catenin is a dual function protein involved in Wnt signaling as well as in cell to cell adhesion [35]. A siRNA knockdown of β-catenin had no effect on 7KCh-induced inflammation (Fig. 6b). Thus, we can conclude that Wnt/β-catenin signaling is not involved in mediating 7KCh-induced inflammation.

**Figure 7:**
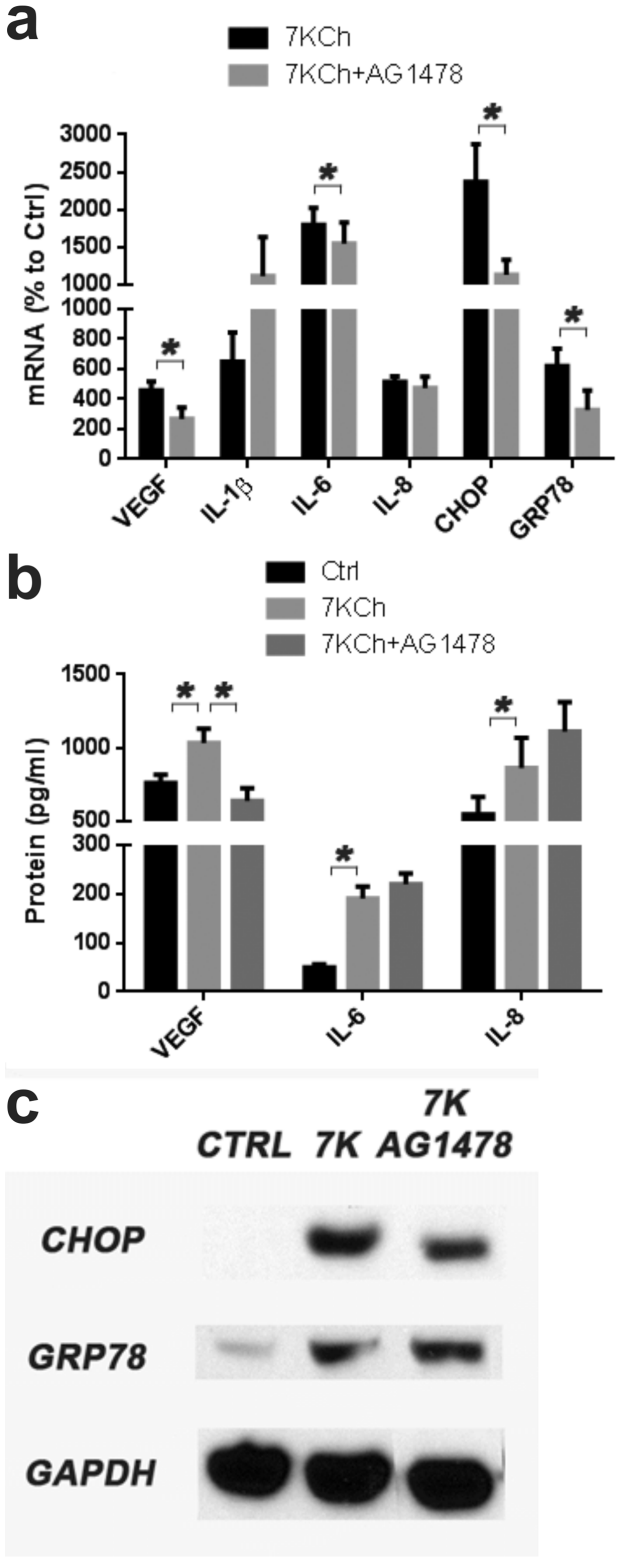
Effects of EGFR inhibition on 7KCh-induced inflammation. ARPE19 cells were treated with 8 µM 7KCh for 24 hr and the mRNA inductions of the inflammatory markers were measured by qRT-PCR. **(a)** Measurements (mean ± s.d., *n* = 3) with and without 5 µM AG1478 (tyrosine kinase inhibitor specific to EGFR). AG1478 suppressed the mRNA induction of VEGF (4.6 to 2.7 fold), CHOP (23.7 to 11.3 fold), and GRP78 (6.2 to 3.3 fold) and a minor but statistically significant reduction in IL-6. It did not have a statistically significant increase on IL-1β and IL-8. **(b)** Measurements of secreted cytokine (mean ± s.d.) in conditioned medium after treatment with 6 µM 7KCh for 48 hr (VEGF, *n* = 3) or 8 µM 7KCh for 24 hr (IL-6 and IL-8, *n* = 4) with and without 5 µM AG1478. AG1478 treatment reduced the 7KCh-induced secretion of VEGF (1035 pg/ml to 638 pg/ml) but had no effect on IL-6 and 8. **(c)** Immunoblot measuring the expression of CHOP and GRP78 in response to AG1478. A slight reduction in CHOP expression was observed but no effect on GRP78. *p<0.05, two-tailed Student's t-test.

It has been previously reported that 7KCh-induced smooth muscle cell migration and proliferation occurs through EGFR and this effect was inhibited by LY294002 and Tyrphostin (AG1478) [17]. We re-examined the involvement of this pathway using AG1478 which is a tyrosine kinase inhibitor with high specificity for EGFR [36]. Treatment AG1478 significantly suppressed the 7KCh-induced mRNA response for VEGF, CHOP and GRP78 (VEGF; 4.6 to 2.7-fold, CHOP; 23.7 to 11.3-fold, GRP78; 6.2 to 3.3). There was a slight but statistically significant effect on IL-6 and no effect on IL-8 (Fig. 7a). There also seems to be a 2-fold increase IL-1β mRNA (Fig. 7a). At the protein level VEGF secretion demonstrated a statistically significant decrease from 1035 to 638 pg/ml but no statistically significant effects were observed for IL-6 and IL-8 (Fig. 7b) and IL-1β was not detected. Immunoblots demonstrated a visible drop in CHOP but no effect on GRP78 (Fig. 7c). The data suggests the 7KCh-induced EGRF signaling may be involved in the induction of IL-1β. Although IL-1β protein was not detected *in vitro*, we have previously observed an increase *in vivo* [9].

### Involvement of the inflammasome

The inflammasome is an important signaling pathway that responds to pathogenic microorganisms and a variety of other stimuli [37]. The mechanisms of activation of the inflammasome are complex [38] and a full explanation is beyond the scope of this study. However, there are several key molecules that can be measured to determine if the inflammasome is activated. These are NLRP3, caspase-1, IL-1β and IL-18 [37], [38].

**Figure 8:**
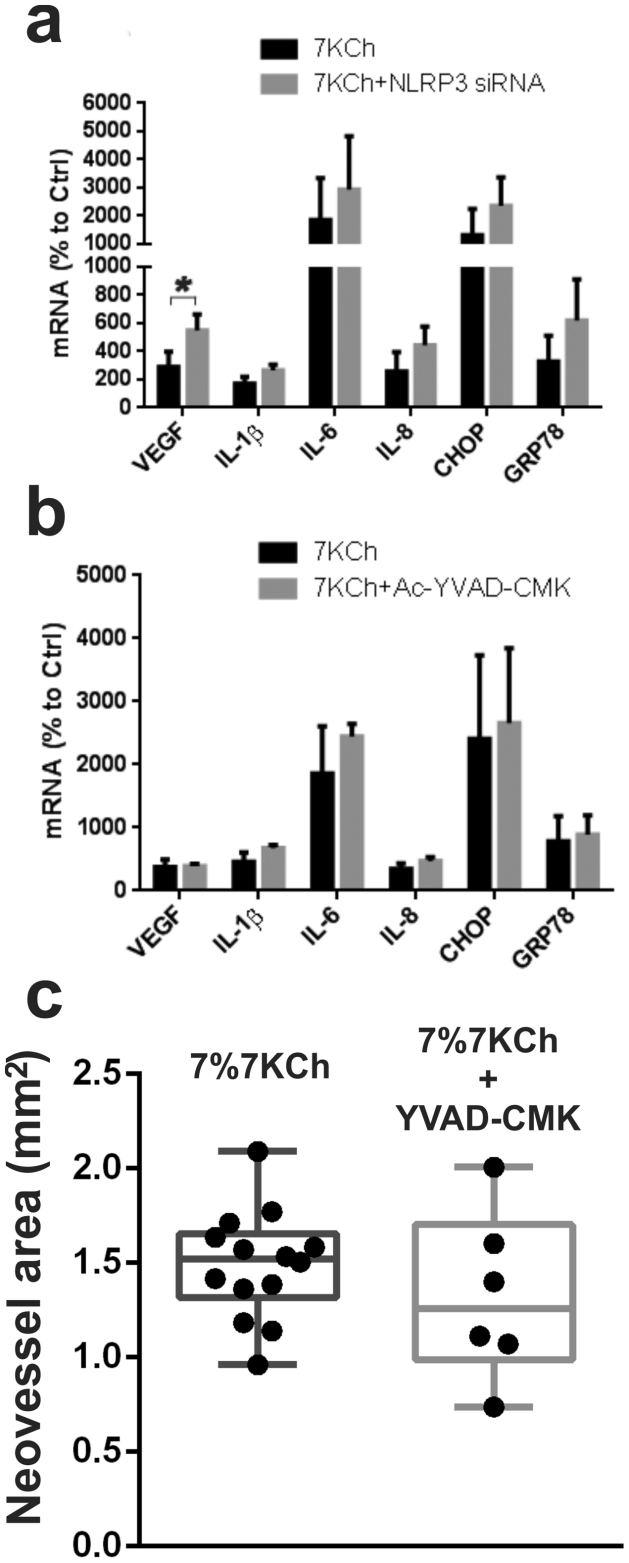
Activation of inflammasome is not involved in 7KCh-mediated inflammation. ARPE19 cells were treated with 8 µM 7KCh for 24 hr and the mRNA inductions of the inflammatory markers were measured by qRT-PCR**. (a)** Measurements (mean ± s.d., *n* = 3) with and without the siRNA knockdown of NLRP3. NLRP3 knockdown caused a slight induction in all of the inflammatory markers but only VEGF was statistically significant (2.9 to 5.5 fold). **(b)** Measurements (mean ± s.d., *n* = 3) with and without t 10 µM Ac-YVAD-CMK (caspase-1 inhibitor). Ac-YVAD-CMK increased IL-6 (18.6 fold to 24.4 fold) but had no statistically significant effect on any of the other markers. *p<0.05, two-tailed Student's t-test. **(c)** Neovessel area measurement (mm^2^) in anterior chamber of the rat eyes in response to 7KCh-containing implants was determined as previously described (9). Implants containing 7% 7KCh (n = 14) and implants containing 7% 7KCh and 10% YVAD-CMK (w/w) (n = 6) were compared. No statistically significant anti-angiogenic effect was observed by YVAD-CMK.

We have found that 7KCh will induce the expression of NLRP3 in ARPE19 cells but no IL1β or IL-18 was detected in the conditioned media cells (data not shown). A siRNA knockdown of NLRP3 increased the mRNA expression of all the inflammatory markers (Fig. 8a) most notably VEGF which nearly doubled (Fig. 8a). Inhibition of caspase-1, the key protein in the inflammasome signaling, with N-acetyl-L-tyrosyl-L-valyl-N-[(1S)-1-(carboxymethyl)-3-chloro-2-oxo-propyl]-L-alaninamide (Ac-YVAD-CMK) [39] also failed to attenuate the 7KCh-induced inflammatory responses (Fig. 8b). Our data suggests that the inflammasome is not directly involved in mediating the initial 7KCh-induced inflammatory responses in ARPE19 cells. However, if the cells are pre-treated with LPS and IL-1α the inflammasome does seem to activate in response to 7KCh [40].

Using our anterior chamber 7KCh implant model we have previously reported that IL-1β levels significantly increased in response to 7KCh [9]. This suggests a possible involvement of the inflammasome response *in vivo* since IL-1β is generally induced via the inflammasome [37]. However, when we inserted implants containing 7% 7KCh and 10% Ac-YVAD-CMK into our anterior chamber rat model [9] no statistically significant anti-angiogeneic reduction was observed (Fig. 8c). Thus, the involvement of the inflammasome in the *in vivo* model needs to be further investigated.

### Involvement of the Toll-like receptor 4 (TLR4)

The TLR4 receptor has been implicated in the inflammation related to atherosclerosis [41]. TLR4 vigorously responds to LPS present in gram negative bacteria, but also responds to numerous other stimuli [42]. Two recent publications are of particular interest since they support and enhance our findings [18], [43]. The first study directly implicates 7KCh in the activation of the TLR4 receptor in placental trophoblasts [18]. The second demonstrated that LY294002 inhibits the production of β-interferon mediated by the TLR4 receptor [43]. This further supports our previous work demonstrating the effectiveness of LY294002 at antagonizing 7KCh-induced inflammation.

**Figure 9:**
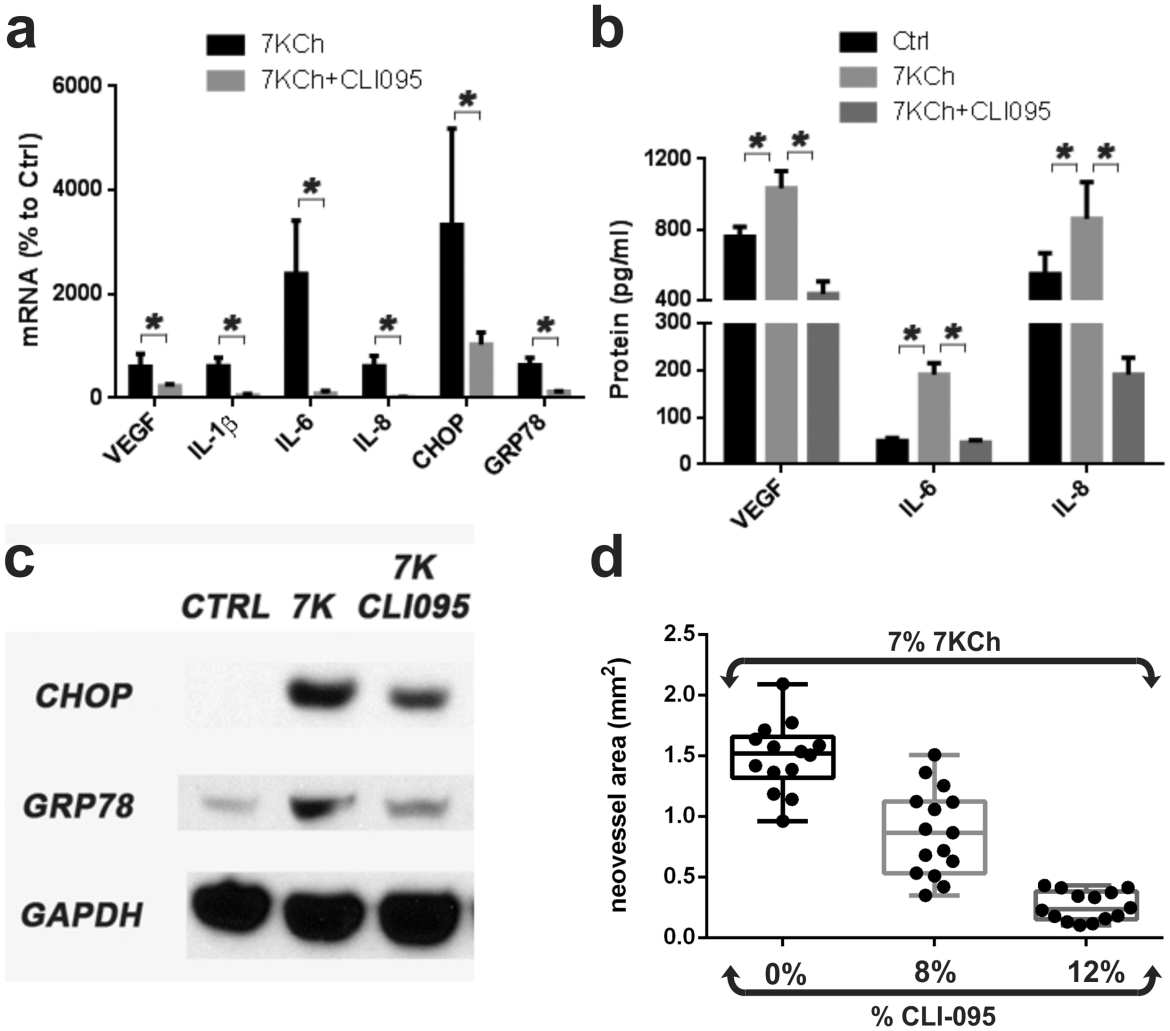
CLI-095 a TLR4 inhibitor significantly suppressed 7KCh-induced inflammation *in vitro* and *in vivo*. ARPE19 cells were treated with 8 µM 7KCh for 24 hr and the mRNA inductions of the inflammatory markers were measured by qRT-PCR. **(a)** Measurements (mean ± s.d., *n* = 4) with and without 10 µM CLI095. CLI-095 significantly reduced the induction of all the inflammatory markers, VEGF (6.0 to 2.3 fold), IL-1β (6.1 to 0.5 fold), IL-6 (23.9 to 0.9 fold), IL-8 (6.1 to 0.1 fold), CHOP (33.3 to 10.3 fold), and GRP78 (6.3 to 1.2 fold). **(b)** Measurements of secreted cytokines (mean ± s.d.) from conditioned in cells treated with 6 µM 7KCh for 48 hr (VEGF, *n* = 3) or 8 µM 7KCh for 24 hr (IL-6 and IL-8, *n* = 4) with and without 10 µM CLI095. CLI095 reduced the secreted VEGF (1035 pg/ml to 436 pg/ml), IL-6 (191 pg/ml to 46 pg/ml), and IL-8 (862 pg/ml to 191 pg/ml) concentrations in conditioned media. IL-1β was not detected. **(c)** Immunoblot demonstrating the reduction in CHOP and GRP78 in response to CLI095 treatment. *p<0.05, two-tailed Student's t-test. **(d)** Comparison of neovessel formation from implants containing 7% 7KCh with 8 and 12% CLI-095. Implants were placed into the anterior chamber of the rat eye as previously described (9). Neovessel areas were measured in mm^2^. The implants containing 8% CLI-095 (n = 14) demonstrated a 41% reduction while the 12% CLI-095 (n = 14) implants demonstrated an 81% reduction in neovessel area.

To follow-up on the TLR4 pathways we used the inhibitor CLI-095 [44]. This inhibitor binds specifically to the TRL4 receptor and prevents it from signaling [45]. CLI-095 reduced the 7KCh-induced inflammation to near basal levels both at the mRNA and protein levels (Fig. 9). The mRNA expression from the four cytokines (VEGF; 6.0 to 2.3-fold, IL-1β; 6.1 to 0.5-fold, IL-6; 23.9 to 0.9-fold, IL-8; 6.1-0.1-fold, 7KCh only, 7KCh+CLI-095, respectively) and the ER stress markers (CHOP; 33.3 to 10.3-fold, GRP78; 6.3 to 1.2-fold) were significantly reduced (Fig. 9a). Similar results were observed at the protein level (VEGF; 1035 to 436 pg/ml, IL-6; 191 to 46 pg/ml, IL-8; 862 to 191 pg/ml, 7KCh only to 7KCh+ CLI-095, respectively) (Fig. 9b). Immunoblots also revealed a significant drop in CHOP and GRP78 (Fig. 9c).

In order to determine if the TLR4 activation by 7KCh seen *in vitro* is also the mechanism *in vivo*, implants containing 7% 7KCh and mixed with CLI-095 (at 8% and 12%) were placed in the anterior chamber of rats and the angiogenesis was measured as previously described [9]. At the 8% CLI-095 concentration CLI-095 inhibited approximately 60% of the angiogenesis and 12% CLI-095 ablated all neovessel formation (Fig. 9d). These results support the *in vitro* results and demonstrate that the majority of the 7KCh-induced inflammation is mediated through the TLR4 receptor.

**Figure 10:**
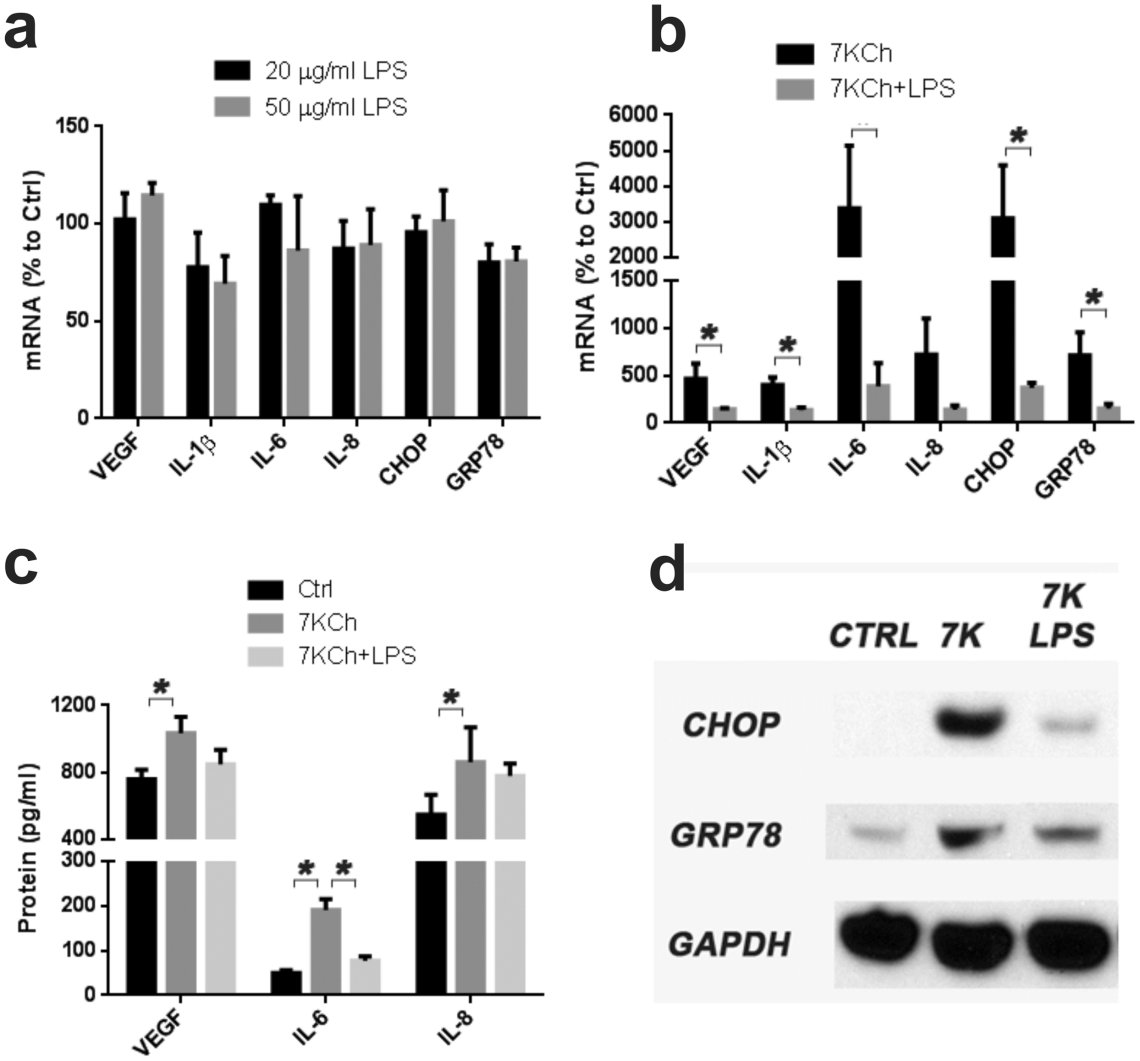
Effect of LPS on 7KCh-mediated inflammation. ARPE19 cells were treated with two concentrations of LPS (20 and 50 µg/ml) for 24 hr and the mRNA inductions of the inflammatory markers measured by qRT-PCR**. (a)** Measurement (mean ± s.d., *n* = 3) of the inflammatory in response to LPS. LPS does not induce any inflammatory response in ARPE19 cells. **(b)** Measurements (mean ± s.d., *n* = 3) in cells treated with 8 µM 7KCh for 24 hr with and without 50 µg/ml LPS. LPS reduced the mRNA induction of all of the inflammatory markers VEGF (4.6 to 1.4 fold), IL-1β (4.0 to 1.4 fold), IL-6 (33.7 to 3.8 fold), IL-8 (7.2 to 1.4 fold), CHOP (31.0 to 3.7 fold), and GRP78 (7.1 to 1.5 fold). **(c)** Measurements of secreted cytokines (mean ± s.d.) from conditioned in cells treated with 6 µM 7KCh for 48 hr (VEGF, *n* = 3) or 8 µM 7KCh for 24 hr (IL-6 and IL-8, *n* = 4) with and without 50 µg/ml LPS. LPS reduced the levels of VEGF (1035 pg/ml to 848 pg/ml) and IL-6 (191 pg/ml to 77 pg/ml) but had no significant effect on IL-8. **(d)** Immunoblot demonstrating that LPS pretreatment significantly reduced the 7KCh-induced expression of CHOP and GRP78. *p<0.05, two-tailed Student's t-test.

In our hands ARPE19 cells do not respond to LPS. ARPE19 cells lack expression of MD-2 and CD14, two key components needed for LPS to activate TLR4 (data not shown). Thus, even extremely high doses of LPS (20 and 50 µg/ml) fail to induce an inflammatory response in ARPE19 cells (Fig. 10a). By contrast, THP-1 and HMVEC cells treated with 10 ng/ml and 100 ng/ml, respectively, show a very robust inflammatory response (data not shown). This demonstrated that our LPS was working properly and that the ARPE19 cell's lack of response was cell-line specific.

To further demonstrate the TLR4 involvement in ARPE19 cells, we measured the effect of LPS (50 µg/ml) on the 7KCh-induced inflammatory response (Fig. 10b). LPS significantly attenuated and/or ablated the 7KCh-induced inflammatory response (Fig. 10b). Similar results were obtained at the protein level for the cytokines VEGF, IL-6 and IL-8 (Fig. 10c) and the ER stress markers CHOP and GRP78 (Fig. 10d). As with the other experiments IL-1β was not detected. This effect suggests that LPS is able to block the TLR4 and block the ability of 7KCh to activate its signaling. As mentioned above, this effect is ARPE19-specific, but nonetheless useful to demonstrate the TLR4 involvement. LPS in our *in vivo* model causes a massive inflammatory response when incorporated into the implants (data not shown).

### 7KCh-induced cell death pathways

**Figure 11:**
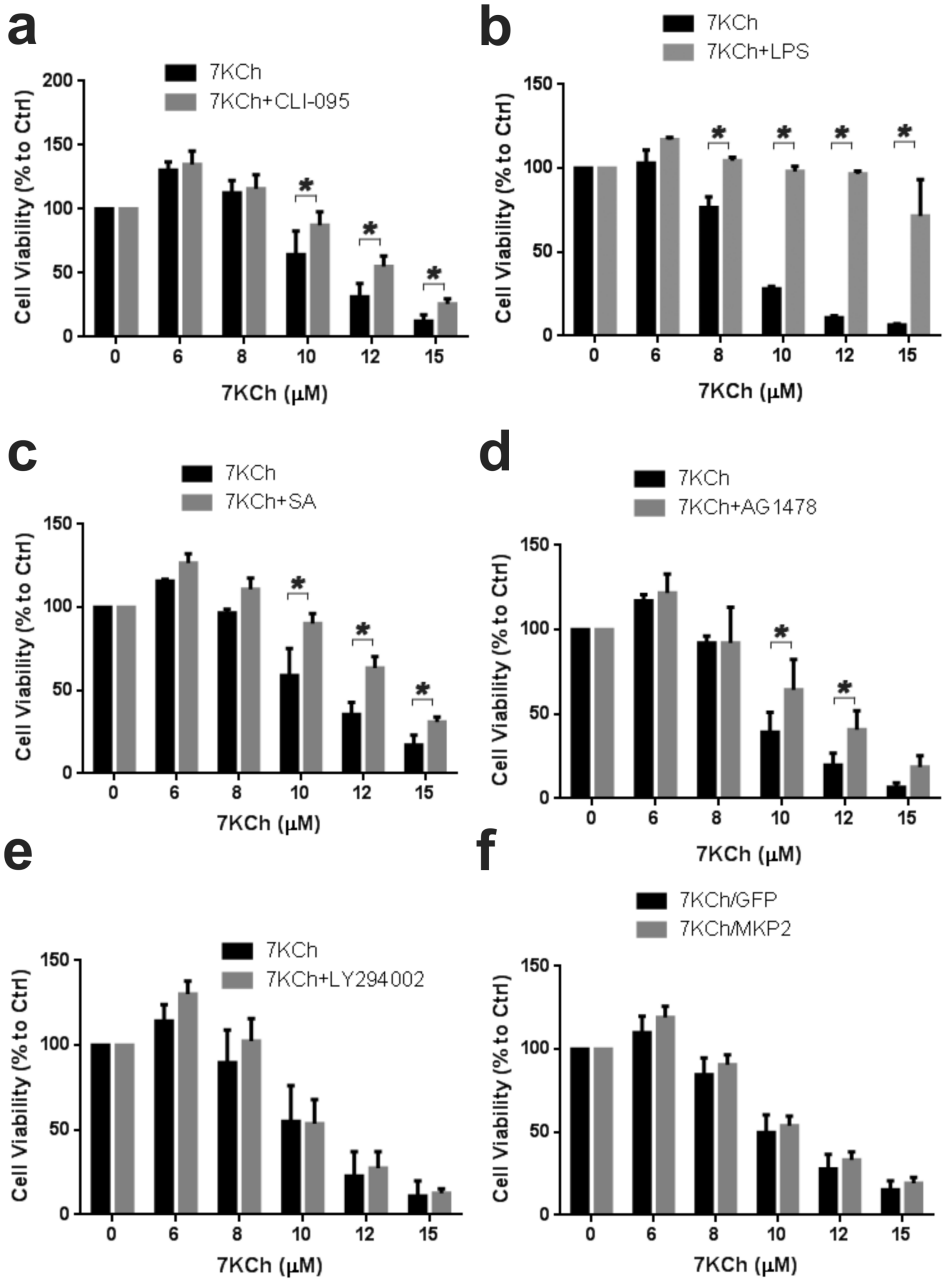
Effects of different inhibitory agents on 7KCh-induced cell death. ARPE19 cells were treated with 6-15 µM 7KCh for 24 hr and the cell viability was measured by determining cellular dehydrogenase activity. **(a)** Viability measurements with and without 10 µM CLI095 (mean ± s.d., *n* = 4). **(b)** Viability measurements with and without 50 µg/ml LPS (mean ± s.d., *n* = 3). **(c)** Viability measurements with and without 1 µM sterculic acid (mean ± s.d., *n* = 4). **(d)** Viability measurements with and without 5 µM AG1478 (mean ± s.d., *n* = 3). **(e)** Viability measurements with and without 10 µM LY294002 (mean ± s.d., *n* = 3). **(f)** Viability measurements with and without MKP2 overexpression (mean ± s.d., *n* = 4). CLI095, LPS, SA, and AG1478 reduced 7KCh-induced cell death while LY294002 and MKP2 overexpression had no effect. *p<0.05, two-tailed Student's t-test.

We have suspected that the cell death pathways for 7KCh are related to NFκB (Fig. 3d). To further investigate the cell death pathways we used CLI-095 and LPS to block the TLR4 function and measure the effect on the cell viability with increasing doses of 7KCh (Fig. 11a and b respectively). CLI-095 inhibition provided partial protection (Fig. 11b) similarly to NFκB inhibition (Fig. 3d). Surprisingly, LPS at 50 µg/ml was able to protect the cells even at the highest concentration of 7KCh (Fig. 11b). Sterculic acid (SA) which we have previously reported to protect cells from 7KCh-induced cell death at 1 µM [19] was included as a positive control (Fig. 11c). The AG1478 inhibitor to EGFR also provided partial protection (Fig. 11d). Interestingly, treatment with LY294002 and MKP2 overexpression which significantly reduced the 7KCh-induced inflammatory responses (Fig. 4a and Fig. 1b, respectively) failed to protect the cells from 7KCh-induced cell death (Fig. 11e and f, respectively). This suggests that the death pathway is influenced by signaling occurring from both upstream and downstream of NFκB. These results also indicate that the cell death pathway is independent from the inflammatory pathway.

### 7KCh-induced ER stress response

**Figure 12:**
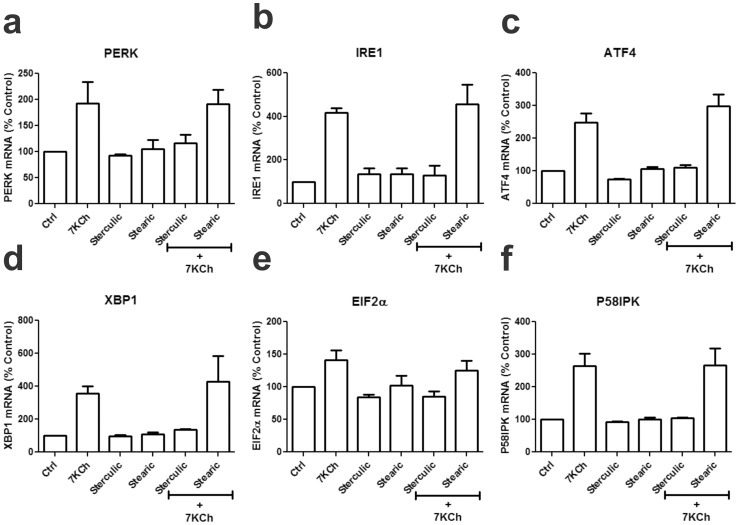
7KCh induces key ER stress markers and SA inhibits this response. ARPE19 cells were treated with 8 µM 7KCh for 24 hr and the mRNA inductions of the inflammatory markers measured by qRT-PCR (mean ± s.d., *n* = 3). **(a)** PERK, **(b)** IRE1, **(c)** ATF4, **(d)** XBP1, **(e)** EIF2a, and **(f)** P58IPK in response to 8 µM 7KCh with and without 1 µM sterculic acid or stearic acid. 7KCh induced the mRNA expression of all key proteins in ER stress pathway. The addition of sterculic acid attenuated the ER stress response (PERK: 1.9 to 1.2 fold, IRE1: 4.2 to 1.3 fold, ATF4: 2.5 to 1.1 fold, XBP1: 3.6 to 1.4 fold, EIF2a: 1.4 to 0.9 fold, P58IPK: 2.7 to 1.0 fold). Stearic acid was used as a negative control.

The 7KCh-induced ER stress response is robust and complex. Measurement of mRNA levels of most of the major ER stress markers by qRT-PCR demonstrated a sharp increase in response to 7KCh treatment (Fig. 12). Yet this seems to occur without PI3K-PIP3-calcium involvement. As mentioned above there is no PI3K involvement (Figs 4, 5) and the use of intracellular calcium chelators failed to attenuate the inflammatory responses (data not shown). NFκB seems to partially mediate these responses since its inhibition was able to attenuate the CHOP and GRP78 inductions (Fig. 3). However, this 7KCh-mediated induction of ER stress markers is not limited to CHOP and GRP78. 7-KCh also induces protein kinase RNA-like endoplasmic reticulum kinase (PERK) (Fig. 12a), Serine/threonine-protein kinase (IRE1) (Fig. 12b), activating transcription factor 4 (ATF4) (Fig. 12c), X-box binding protein 1 (XBP1) (Fig. 12d), eukaryotic initiation factor 2 alpha (IEF2α) (Fig. 12e) and p58ipk (inhibitor of interferon-induced double-stranded RNA-activated protein kinase) (Fig. 12f). The eukaryotic initiation factor 2 (eIF2α) and IRE1are also phosphorylated in response to 7KCh (data not shown). SA was able to attenuate and/or ablate all of the ER stress-related 7KCh-induced inflammatory responses (Fig. 12). These results suggested two things: 1) SA is likely a kinase inhibitor (more on this below) and 2) other, yet unidentified kinases may be involved in initiating the ER stress response.

The ER stress response also suggested a possibility for identifying the 7KCh-induced cell death pathway mentioned above. ATF4 which is known to be also activated by TLR4 [46] and is known to mediate CHOP expression [47], may be a possible mediator the 7KCh-induced cell death pathway. CHOP is well-known to be involved in ER stress induced apoptosis [47].

**Figure 13:**
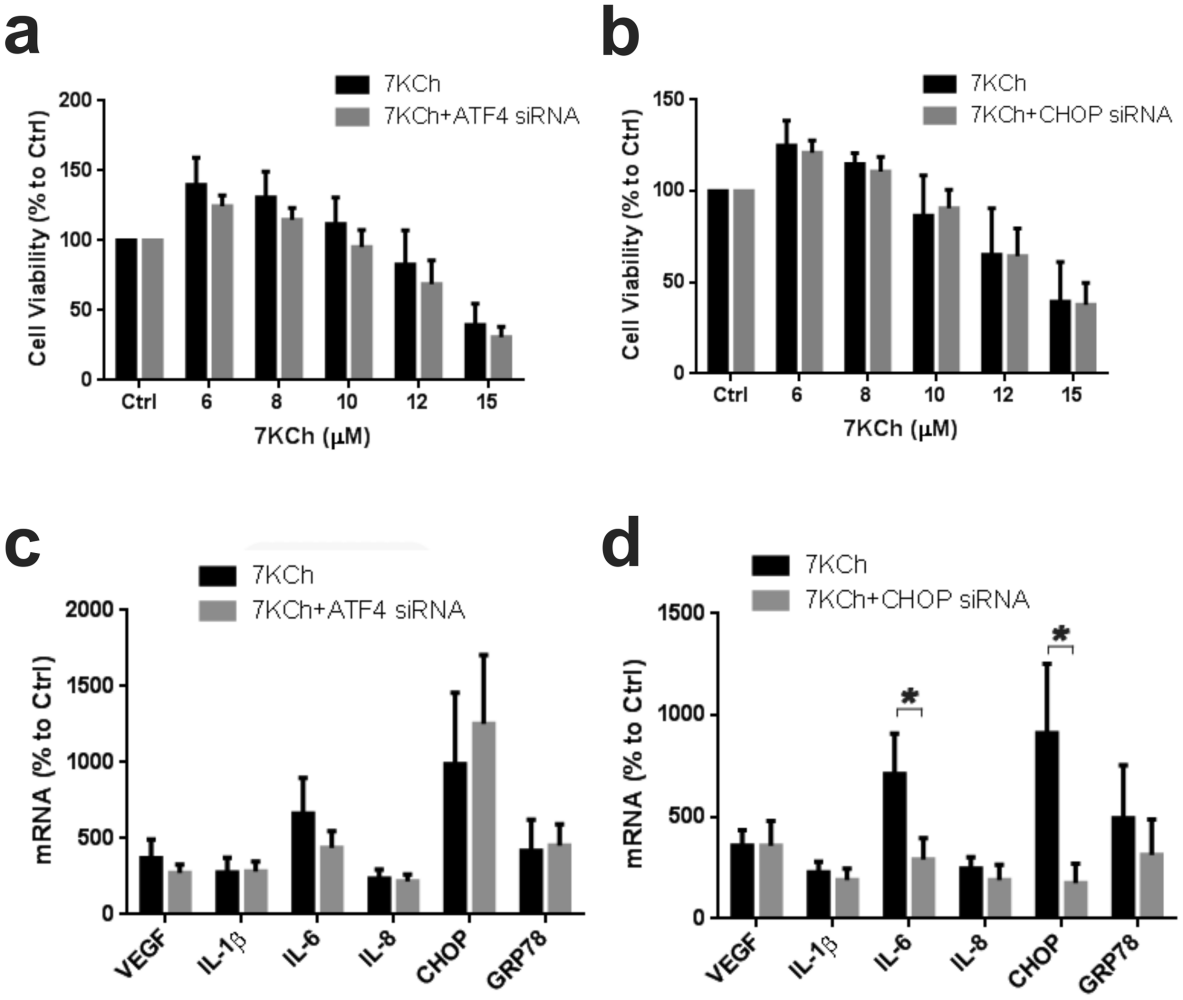
siRNA knockdown of ATF4 or CHOP had no effect on 7KCh-induced cell death but reduced IL-6 levels. ARPE19 cells were treated with 6-15 µM 7KCh for 24 hr and the cell viability was measured by determining cellular dehydrogenase activity (mean ± s.d., *n* = 4) with and without knockdown of **(a)** ATF4 and **(b)** CHOP by siRNAs. ARPE19 cells were treated with 8 µM 7KCh for 24 hr and the mRNA inductions were measured by qRT-PCR (mean ± s.d., *n* = 3). **(c)** Measurement with and without siRNA knockdown of ATF4 (mean ± s.d., *n* = 3) **(d)** Measurement with and without siRNA knockdown of CHOP (mean ± s.d., *n* = 3). Both ATF4 and CHOP knockdowns reduced the 7KCh-induced IL-6 expression but it was only statistically significant with CHOP (7.1 to 2.9 fold). *p<0.05, two-tailed Student's t-test.

To further investigate this potential pathway siRNAs were used to knockdown ATF4 and CHOP (Fig. 13). The knockdown of both ATF4 and CHOP failed to attenuate the 7KCh-induced cell death (Fig 13 a,b). The siRNA knockdown of ATF4 also had no statistically significant effect on any of the inflammatory markers (Fig. 13c). The siRNA knockdown of CHOP did cause a 60% decrease IL-6 induction (Fig. 13d). The siRNA, as expected, reduced CHOP expression by 81% (Fig. 13d). This suggests that the 7KCh-induced cell death pathway is not mediated by ATF4/CHOP or the observed ER stress response.

### TLR4 signaling pathways

The TLR4 receptor is known to signal via four adaptor proteins which work in pairs [48]. The myeloid differentiation primary response gene-88 (MyD88) partners with toll-interleukin-1 receptor (TIR) domain containing adaptor protein (TIRAP) and the TIR domain-containing adapter protein (TRIF) partners with TRIF-related adaptor molecule (TRAM) [48]. The MyD88/TIRAP signaling involves the activation of interleukin-1 receptor-associated kinase-4 (IRAK4) with subsequent phosphorylation of IRAK1 which eventually activates NFκB via the iκB kinases, α, β and γ complex (IKKα, IKKβ and IKKγ). The TRIF/TRAM signaling activates the receptor interacting protein 1 kinase (RIP1) which phosphorylates the IKKε/TBK1 complex which then phosphorylates iκB and activates the NFκB complex [48].

**Figure 14:**
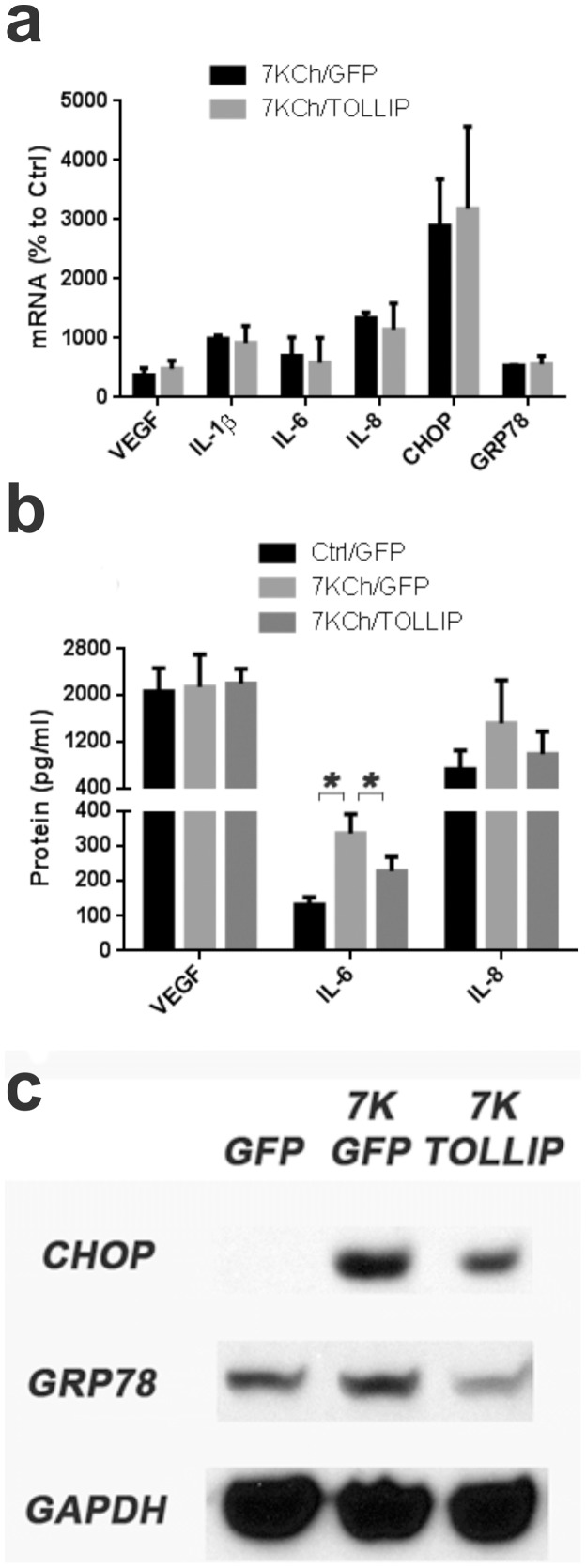
Effect of TOLLIP overexpression on 7KCh-induced inflammation. ARPE19 cells were transduced with a commercially available adenovirus expressing TOLLIP (an IRAK4 inhibitor). After transduction cells were treated with 8 µM 7KCh for 24 hr and the inflammation markers measured by qRT-PCR**. (a)** Measurements (mean ± s.d., *n* = 2) with and without the overexpression of TOLLIP. TOLLIP overexpression did not alter the mRNA induction of any of the inflammatory markers. **(b)** Measurements of secreted cytokines (mean ± s.d.) from conditioned in cells treated with 6 µM 7KCh for 48 hr (VEGF, *n* = 3) or 8 µM 7KCh for 24 hr (IL-6 and IL-8, *n* = 4) with and without TOLLIP overexpression. TOLLIP overexpression had a statistically significant reduction in IL-6 expression (337 pg/ml to 228 pg/ml) but no statistically significant effect on VEGF or IL-8. **(c)** Immunoblot demonstrating the protein expression CHOP and GRP78. TOLLIP overexpression caused a small decrease in CHOP and GRP78 protein expression. GFP overexpression was used as control. *p<0.05, two-tailed Student's t-test.

**Figure 15:**
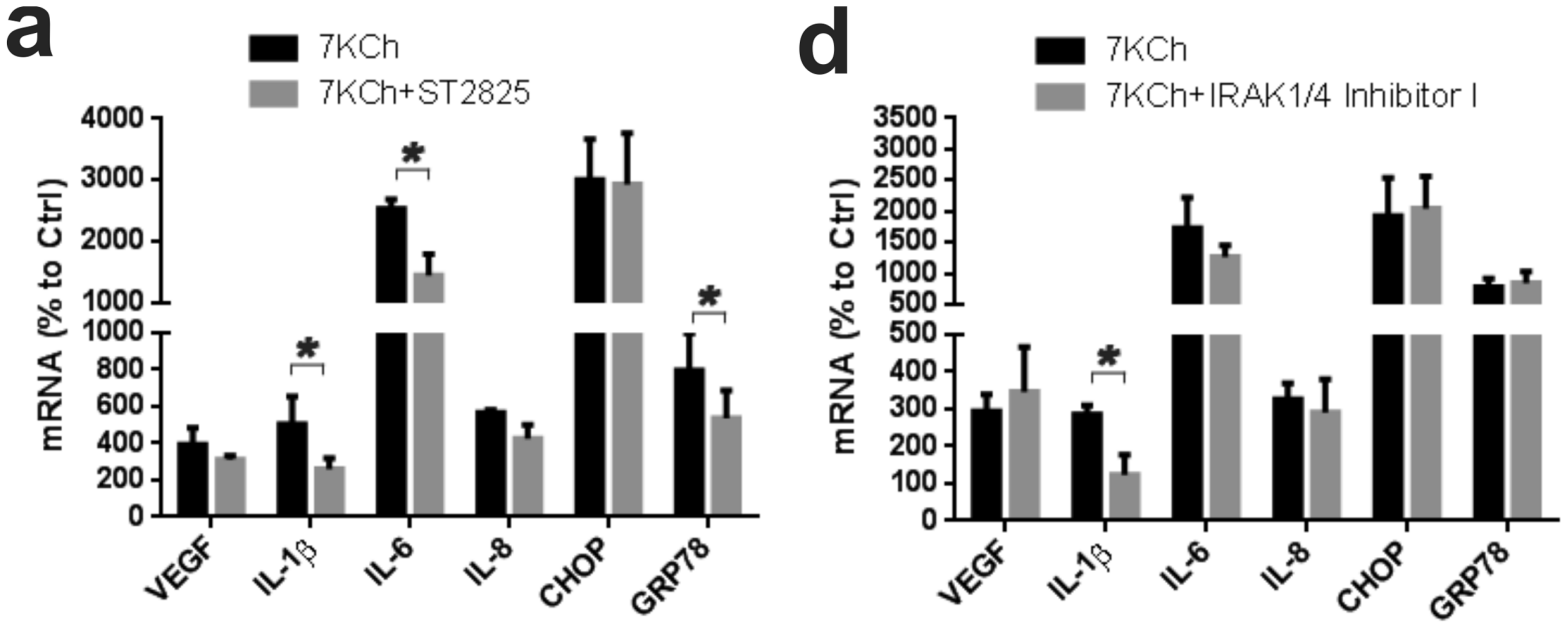
Inhibition of TIRAP/MYD88 side of the TRL4 receptor. ARPE19 cells were treated with 8 µM 7KCh for 24 hr and the mRNA inductions of the inflammatory markers measured by qRT-PCR (mean ± s.d., *n* = 3). **(a)** Measurements with and without 10 µM ST2825 (MyD88 inhibitor). ST2825 suppressed the induction of IL-1β (5.0 to 2.6 fold), IL-6 (25.3 to 14.5 fold), and GRP78 (8.0 to 5.4 fold) but had no effect on VEGF, IL-8 and CHOP. **(b)** Measurement with and without 5 µM IRAK1/4 inhibitor I. The IRAK1/4 inhibitor I suppressed the induction of IL-1β (2.9 to 1.2 fold) but had no effect on the other markers. *p<0.05, two-tailed Student's t-test.

To determine which of these two pathways (MyD88-TIRAP or TRIF-TRAM) signals in response to 7KCh treatment, Toll interacting protein (TOLLIP) was overexpressed in ARPE19 cells by transducing the cells with a TOLLIP overexpressing adenovirus. TOLLIP inhibits the activation of IRAK1 by IRAK 4. The TOLLIP overexpression had no effect on the induction of the mRNAs of the inflammatory markers (Fig. 14a). TOLLIP had a small but statistically significant effect on the IL-6 protein expression (Fig. 14b) and ER stress markers CHOP and GRP78 (Fig. 14c). This suggested that little signaling is occurring through MyD88/TIRAP. However, since these inflammatory pathways are complex and often redundant, we performed some additional experiments to further define the MyD88/TIRAP adaptor pair signaling. The MyD88 inhibitor ST2825 [49] and the IRAK1/4 inhibitor I [50] were used to suppress their activity. The ST2825 inhibitor attenuated the induction of IL-1β (approximately 48%) and GRP78 (43%) mRNA levels (Fig. 15a). The IRAK1/4 inhibition also had a significant effect on IL-1β (58% reduction) but no statistically significant effect on any of the others (Fig 15b). This suggests that perhaps most of the IL-1β signaling is occurring via the MyD88/TIRAP but the response by the other markers seems to occur via TRIF/TRAM.

**Figure 16:**
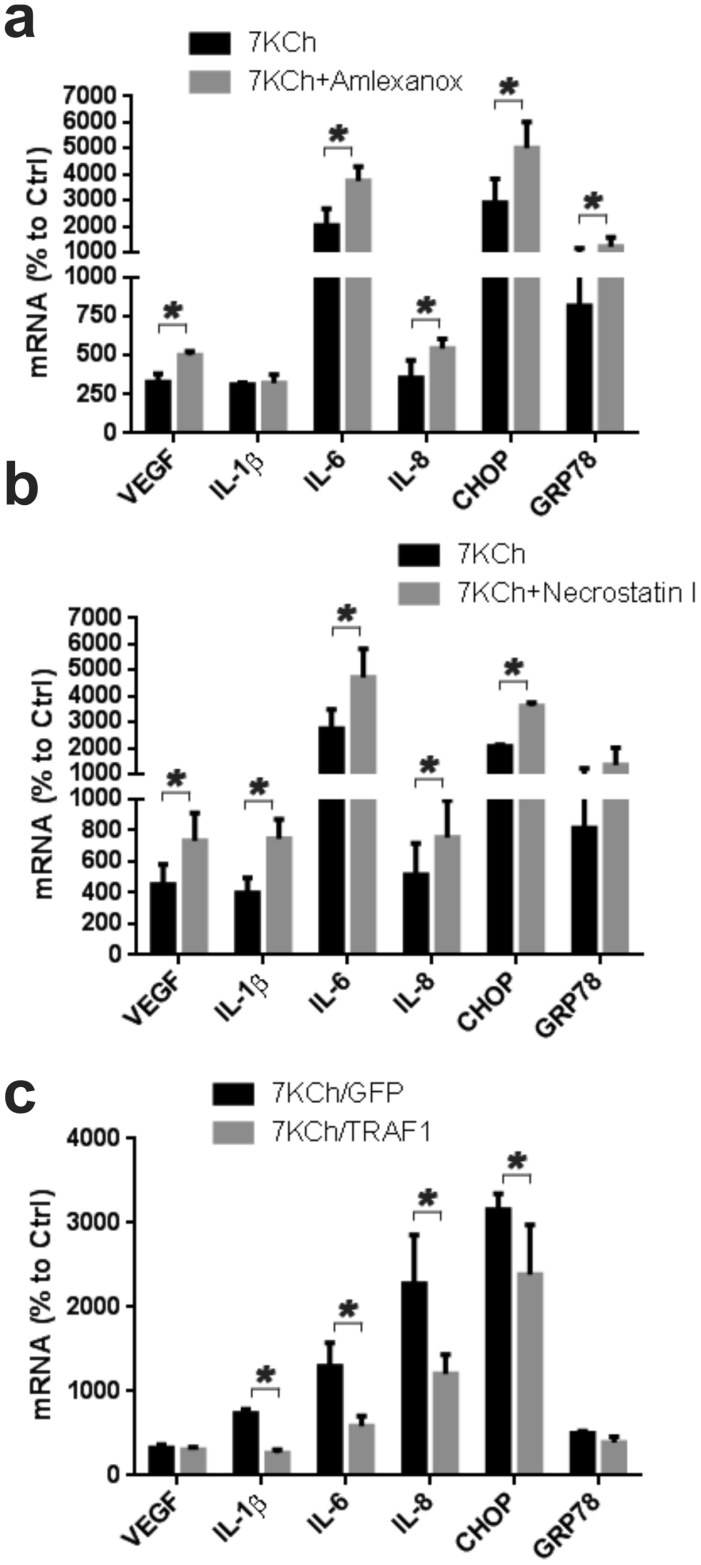
Inhibition of the TRIF/TRAM side of the TRL4 receptor. ARPE19 cells were treated with 8 µM 7KCh for 24 hr and the mRNA inductions of the inflammatory markers measured by qRT-PCR (mean ± s.d., *n* = 3). (a) Measurement with and without 10 µM Amlexanox (inhibitor of IKKε/TBK1 complex). Amlexanox enhanced the expression VEGF (3.3 to 5.0 fold), IL-6 (20.2 to 37.6 fold), IL-8 (3.5 to 5.4 fold), CHOP (28.9 to 50.1 fold), and GRP78 (8.2 to 12.2 fold). **(b)** Measurements with and without 10 µM Necrostatin I (RIP1 inhibitor). Necrostatin I enhanced the induction of VEGF (4.5 to 7.3 fold), IL-1β (4.0 to 7.5 fold), IL-6 (27.3 to 47.1 fold), IL-8 (5.1 to 7.5 fold), CHOP (20.7 to 36.1 fold), and GRP78 (8.2 to 13.5 fold). TRAF1 (a TRIF negative regulator) was overexpressed by transducing the cells with a commercially available adenovirus. **(c)** Measurements (mean ± s.d., *n* = 4) with and without TRAF1 overexpression. TRAF1 overexpression suppressed the induction of IL-1β (7.4 to 2.7 fold), IL-6 (13.0 to 5.9 fold), IL-8 (22.8 to 12.0 fold), and CHOP (31.6 to 23.9 fold) but had no effect on VEGF and GRP78. GFP overexpression was used as control. *p<0.05, two-tailed Student's t-test.

The TRIF/TRAM pathway also signals through NFκB but via RIP1, tumor necrosis factor receptor associated factor 6 (TRAF6), TANK binding kinase (TBK1) and inhibitor of nuclear factor kappa-B kinase subunit epsilon (IKKε). Amlexanox, a IKKε/TBK1 inhibitor [51] did not attenuate the inflammatory response but potentiated it instead. Statistically significant increases in VEGF, IL-6, CHOP and GRP78 were observed (Fig. 16a). Necrostatin, and inhibitor to the RIP1 kinase [52] also failed to attenuate the inflammatory response and instead caused statistically significant increases in all of the inflammatory markers (Fig. 16b). Overexpression (by transducing with an adenovirus) of TNF-associated factor-1 (TRAF1), a negative regulator of TRIF [53], demonstrated a statistically significant attenuation the 7KCh-induced inflammatory response for IL-1β, IL-6, IL-8 and CHOP but had no effect of VEGF and GRP78 (Fig. 16c). This supports prior observation suggesting that most of the signaling is through TRIF/TRAM. However, this signaling seems to be occurring via an atypical set of downstream intermediates, most likely unidentified kinases.

### Sterculic acid (SA) binds to various kinases that signal downstream of TLR4

The above results suggest that most of the TLR4 signaling is occurring through TRIF/TRAM but via some irregular intermediaries. This prompted us to take a closer look at SA which is a very potent antagonist of 7KCh-induced inflammation [19], and ER stress (Fig. 12). We used SA to find other signaling kinases downstream of TRIF/TRAMperforming a Kinome*scan*. This is a competitive binding-based screening of 395 individual kinases performed as a service by DiscoverRx (www.discoverx.com). The Kinome*scan* found that SA at 5 µM does not strongly bind to any of the kinases tested. However, it does have some affinity for 16 different kinases. The results of the top scoring kinases (with scores below 60% of control or 40% inhibition) are shown in Table 1. The highest binding interference was with the carboxy terminal kinase domain (CTKD) of the p90 ribosomal kinase 3 (Table 1). However, SA also interfered with the binding to the CTKD of the other three p90 ribosomal kinases (RSKs) (Table 1). Moreover, the CTKD of the RSKs is homologous to the calcium/calmodulin-dependent protein kinase superfamily family (CAMK) [54]. The majority of the kinases (10 out of 16) that interacted with SA belong to the CAMK group (Table 1). Thus, it would be reasonable to assume that SA has some affinity to the kinase domains of these enzymes. Since these binding assays do not measure activity, and all four RSKs were affected, we further investigated their involvement in mediating the 7KCh-induced inflammation and cell death with specific inhibitors.

**Table 1:** Results from Kinomescan.

Kinase	Binding (% of Control)	Full name	Group
CAMK1	40	Calcium/calmodulin-dependent protein kinase type 1	CAMK
CAMK2G	57	Calcium/calmodulin-dependent protein kinase type 2	CAMK
CAMKK2	58	Calcium/calmodulin-dependent protein kinase kinase 2	CAMK
DAPK1	51	Death-associated protein kinase 1	CAMK
DYRK1B	49	Dual specificity tyrosine-phosphorylation-regulated kinase 1B	CMCG
IKKα	51	IκB kinase α	other
IKKβ	53	IκB kinase β	other
MARK3	44	MAP/microtubule affinity-regulating kinase 3	CAMK
MST3	58	mammalian Sterile20-related kinase 3	STE
PHKG1	59	Phosphorylase b kinase gamma catalytic chain, skeletal muscle isoform	CAMK
PNK1	53	Polynucleotide kinase 1	AGC
RSK1 (Kin. Dom-2 C-term)	52	p90 ribosomal S6 kinase 1	CAMK
RSK2 (Kin. Dom-2 C-term)	45	p90 ribosomal S6 kinase 2	CAMK
RSK3 (Kin. Dom-2 C-term)	34	p90 ribosomal S6 kinase 3	CAMK
RSK4 (Kin. Dom-2 C-term)	58	p90 ribosomal S6 kinase 4	CAMK
TLK1	59	Serine/threonine-protein kinase tousled-like 1	other

List of kinases that demonstrated 40% or more of competitive inhibition in the KinomeScan assay. For details go to http://www.discoverx.com/technologies-platforms/competitive-binding-technology/kinomescan-technology-platform.

**Figure 17:**
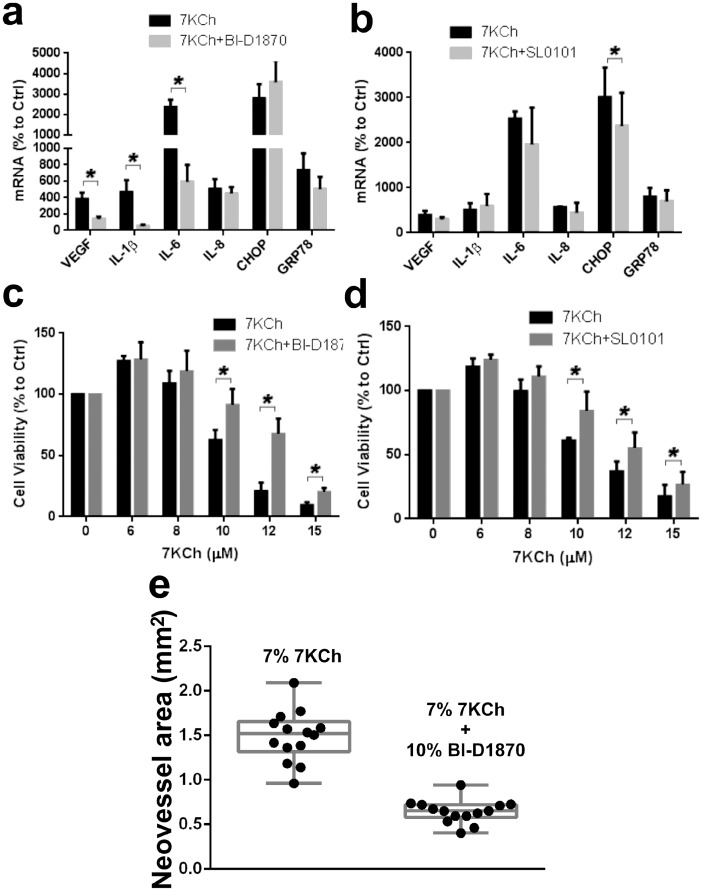
Effect of RSK inhibitors (BI-D1870 and SL0101) on 7KCh-induced inflammation and cell death. ARPE19 cells were treated with 8 µM 7KCh for 24 hr and the mRNA inductions of the inflammatory markers measured by qRT-PCR (mean ± s.d., *n* = 3). **(a)** Measurements with and without 10 µM BI-D1870 (RSK1-4 inhibitor) and **(b)** Measurements with and without 10 µM SL0101 (RSK1/2 inhibitor). BI-D1870 significantly suppressed the induction of VEGF (3.8 to 1.4 fold), IL-1β (4.7 to 0.5 fold), and IL-6 (23.7 to 5.9 fold). SL0101 only caused a slight suppression in the induction of CHOP (30.1 to 23.7 fold). ARPE19 cells were treated with 6-15 µM 7KCh for 24 hr and the cell viability was measured by determining cellular dehydrogenase activity (mean ± s.d., *n* = 4). **(c)** Cell viability with and without 1 µM BI-D1870 (mean ± s.d., *n* = 3) **(d)** Cell viability with and without 10 µM SL0101 (mean ± s.d., *n* = 4). Both BI-D1870 and SL0101 significantly protected the ARPE19 cells from 7KCh-induced cell death. *p<0.05, two-tailed Student's t-test. **(e)** Inhibition of angiogenesis in the anterior chamber rat model (9) with implants containing 7% 7KCh(n = 14) with and without 10% BI-D1870 (n = 14). BI-D1870 caused an 81% inhibition in the angiogenesis.

The RSKs inhibitors BI-D1870 [55] and SL0101 [56] were tested to see if they suppress the 7KCh-induced inflammation and cell death (Fig. 17). The BI-D1870 significantly attenuated the VEGF, IL-1β and IL-6, but had no effect on IL-8, CHOP and GRP78 (Fig. 17a). SL0101 only caused a slight but statistically significant decrease in CHOP (Fig. 17b). However, both BI-D1870 and SL0101 provided significant protection from 7KCh-induced cell death (Fig 17c, d). In our anterior chamber *in vivo* model [9], implants containing 7% 7KCh and 10% BI-D1870 had 66% less neovessel formation that 7KCh alone (Fig.17e). Implants containing 7% 7KCh and 10% SL0101 did not have any effect on reducing the 7KCh-induced angiogenesis (data not shown). This strongly suggests that there is a significant difference between the inflammatory and cell death pathways and both pathways are mediated by RSKs.

The differences between BI-D1870 and SL0101 have only been studied for RSK1 and 2 [57]. BI-D1870 is a more potent inhibitor of RSK1/2 and is also a potent inhibitor of polo-like kinase 1 (PLK1). Thus, SL0101 is more specific to RSK1/2 and has no effect on PLK1 [57]. This implicates RSK1/2 in the cell death pathway, and by default RSK3/4 in the inflammatory pathway since BI-D1870 is also a potent inhibitor of all RSKs.

### Involvement of Suppressor of cytokine signaling (SOCS)

**Figure 18:**
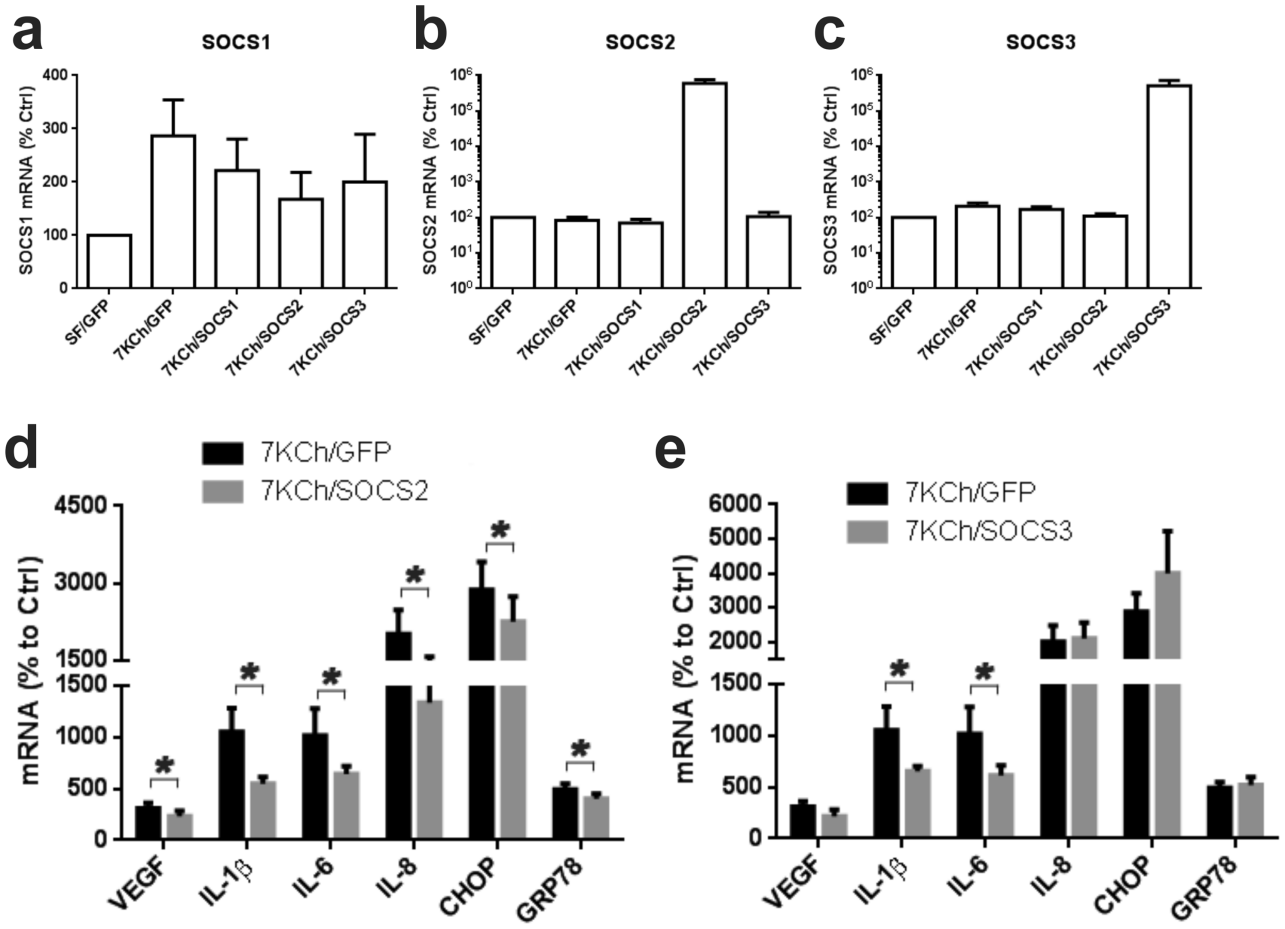
SOCS overexpression and its effect on 7KCh-induced inflammation. ARPE19 cells were transduced with commercially available adenoviruses coding for SOCS1-3 and GFP. The adenovirus coding for GFP was used as control. Cells were then treated with 8 µM 7KCh for 24 hr and the SOCSs mRNA levels measured by qRT-PCR. The Y-axis in in log base 10. **(a)** Measurement of SOCS1 after transduction SOCS1-3 viruses with and without 7KCh treatment. 7KCh induced SOCS1 mRNA but the transduction with the SOCS1 virus did not have a significant effect on the mRNA levels. **(b)** 7KCh had no effect on the induction of SOCS2 but the virus increased the mRNA levels by 3-fold. **(c)** 7KCh had no effect on the induction of SOCS3 but the virus increased the mRNA levels by 3-fold. ARPE19 were transduced cells with adenoviruses for SOCS2 and 3 then treated with 8 µM 7KCh for 24 hr. The mRNA inductions of the inflammatory markers were measured by qRT-PCR (mean ± s.d., *n* = 4). **(d)** Measurements with and without SOCS2 overexpression. SOCS2 overexpression suppressed the 7KCh induction of VEGF (3.1 to 2.3 fold), IL-1β (10.5 to 5.5 fold), IL-6 (10.2 to 6.4 fold), IL-8 (20.1 to 13.3 fold), CHOP (28.7 to 22.6 fold), and GRP78 (4.9 to 4.0 fold). **(e)** Measurements with and without SOCS3 overexpression. Overexpression of SOCS3 suppressed the 7KCh induction of IL-1β (10.5 to 6.5 fold) and IL-6 (10.2 to 6.1 fold), but had no effect on the other markers. *p<0.05, two-tailed Student's t-test.

SOCS are a family of proteins that are generally known for their inhibition of the Janus kinase-signaling and activator of transcription pathways (JAK/STAT) [58], [59]. SOCS1-3 are also known to inhibit TLR signaling by binding to various signaling molecules including MyD88, TRAFs, IRAKs, TAK1 and NFκB [60]. To determine if SOCSs were involved in regulating 7KCh-induced inflammation, SOCS1-3 were overexpressed in ARPE19 cells by transducing with replication negative adenoviruses. The effects of 7KCh on the transduced cells were determined for each SOCS (Fig. 18 a-c, log scale). A GFP overexpressing virus was used as control. 7-KCh treatment caused a minor increase in SOCS1 mRNA (Fig. 18a) but the SOCS1 overexpressing virus did not cause a statistically significant increase in SOCS1 (Fig. 18a). The SOCS1 induction was minor as compared with SOCS2 and 3 (Fig. 18a-c, see Y-axis).7KCh treatment did not cause an increase in SOCS2 levels, but the SOCS2 overexpressing virus increased the SOCS2 mRNA by 6000-fold (Fig. 18b). Similar results were observed for the SOCS3 virus (5200-fold) (Fig. 18c). The SOCS1 overexpressing virus was not used in any of the follow-up experiments. The SOCS2 overexpression caused a statistically significant decrease in all of the inflammatory markers (Fig. 18d) while overexpression of SOCS3 only decreased IL-1β and IL-6 (Fig. 18e). This provided us additional evidence of the involvement of the TLR4 and EGFR pathways.

## Discussion

7-KCh has been associated with numerous age-related and neurodegenerative diseases [10], [11], [61], [62]. It is not toxic when ingested since it can be detoxified by the liver [63], [64]. The toxicity associated with 7KCh is only of consequence when it forms *in situ* in peripheral tissues that lack the detoxifying mechanisms present in the liver. 7-KCh is generally found where lipoprotein deposits accumulate, usually in the cardiovascular system [2]–[4] and in the back of the eye [5]. Chronic systemic low-grade inflammation is suspected as being a cause of aging and age-related diseases [65]. Hence, understanding the molecular mechanism of inflammation caused by 7KCh, a molecule that forms and accumulates as a consequence of age-related oxidation, may prove to be extremely important for developing small molecule therapies to attenuate this inflammation and delay the onset of chronic age-related diseases.

**Figure 19:**
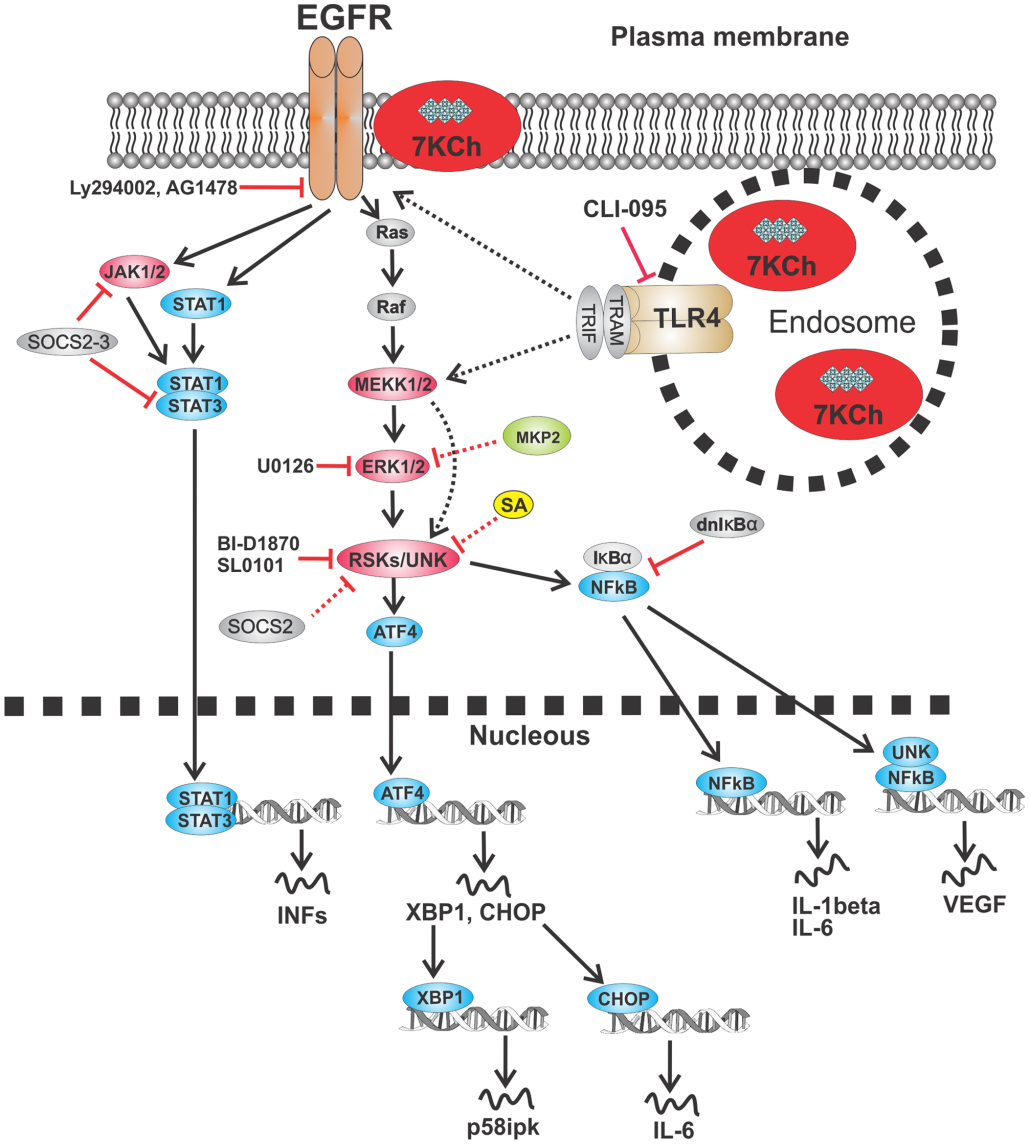
Schematic of EGFR signaling and the possible interconnections with the TLR4 receptor. The solid arrows and lines are published interactions dotted are speculative and/or based on interaction in Table 2.

**Figure 20:**
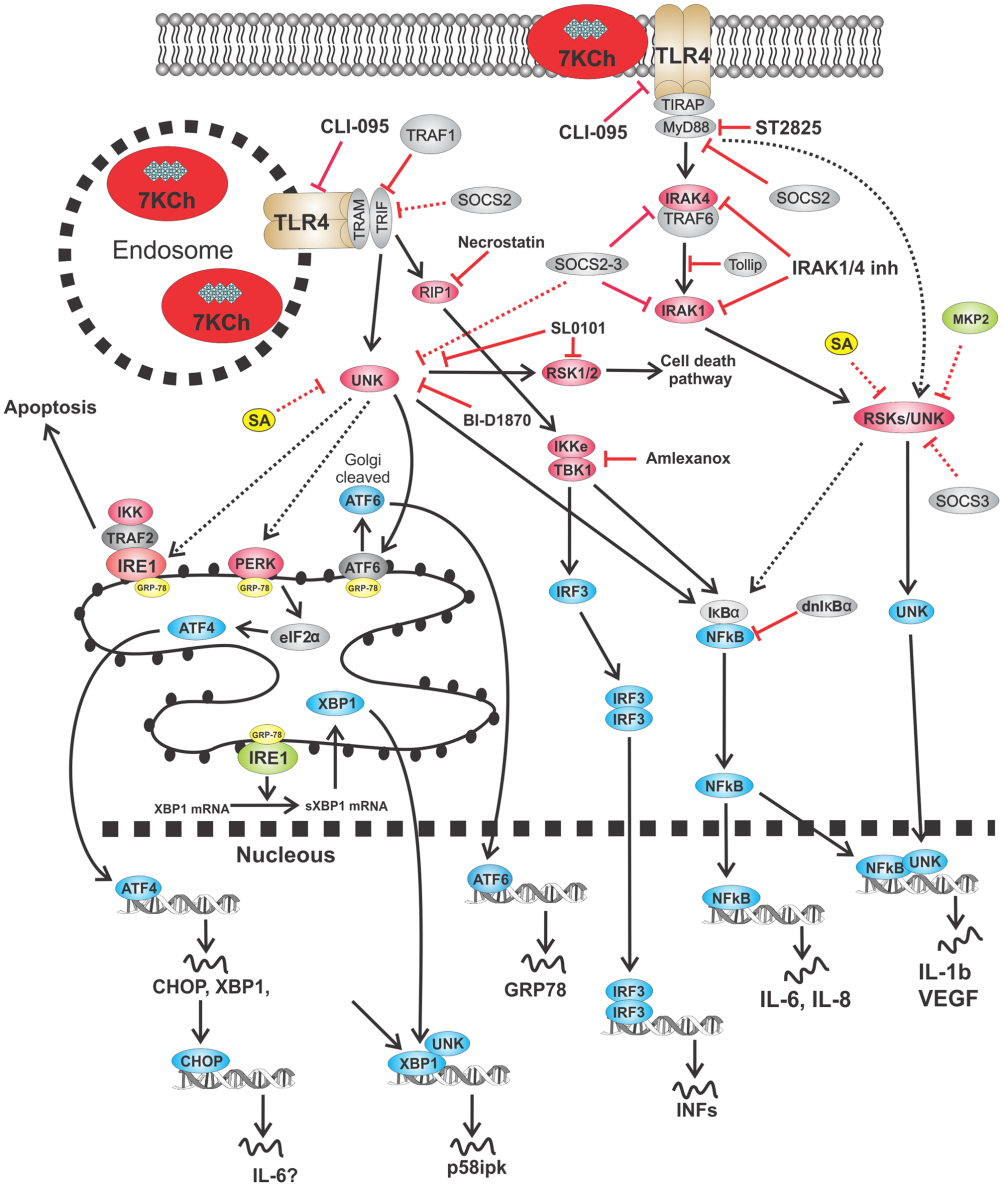
Schematic of TLR4 signaling. The solid arrows and lines are published interactions dotted are speculative and/or based on interaction in Table 2.

The inflammatory pathways involved in 7KCh-induced inflammation are complex. A summary of all of the interactions we have mentioned in this study are summarized in Table 2 and schematics are provided in figures 19 and 20. The 7KCh-induced inflammatory responses have been investigated by numerous laboratories and have resulted in different and often contradictory results [7]–[18]. We have systematically examined the different published mechanisms for 7KCh-induced inflammation and have excluded several of them as being involved in our *in vitro* (ARPE19 cells) and in our *in vivo* anterior chamber 7KCh implant angiogenesis model [9]. In our systems the MAPKs (Fig. 2), PI3K (Fig. 4, 5), Akt (Fig. 4, 5), CK2, Wnt/β-catenin (Fig. 6) do not seem to be involved. The involvement of the inflammasome is questionable. In our *in vitro* system it does not appear to be directly involved since siRNA knockdown of NLRP3 and caspase-1 inhibition have no effect (Fig. 8a, b). However, we previously reported high induction of IL-1β in the aqueous humor in our *in vivo* model [9]. Since IL-1β mostly signals through the inflammasome [37], [38], we suspected it may be involved *in vivo*. Incorporation of the caspase-1 inhibitor YVAD-CMK into the 7KCh-containing implants did not inhibit angiogenesis (Fig. 8c). This may not be conclusive evidence of lack of inflammasome response since YVAD-CMK is a water soluble peptide and may have diffused from the implant before the macrophages arrived and respond to 7KCh. However, implants containing 15% silica (a known inflammasome activator, [37], [38] caused only minimal inflammation in our *in vivo* rat anterior chamber model (data not shown). This suggests that the inflammasome is unlikely to be the primary responder to 7KCh-induced inflammation.

**Table 2:** List of inhibitors and their effects on 7KCh-induced inflammation.

INHIBITOR	Target/Pathway	Conc (µM)	*in vivo*	VEGF	IL-1β	IL-6	IL-8	CHOP	GRP78	Cell death
MKP2 overexpression	JNK, ERK, p38MAPK	N/A	N/A	↓58%	↓95%	↓75%	↓81%	↓45%	↓59%	None
SP600125	JNK	5	N/A	↔	↔	↑42%	↔	↑25%	↔	ND
UO126 (ERK1/2)	ERK1/2 EGFR	10	N/A	↔	↔	↔	↔	↔	↔	ND
SB203580	p38MAPK	10	N/A	↔	↑64%	↔	↑23%	↔	↔	ND
dnIκBα overexpression	NFκB	N/A	N/A	↓29%	↓83%	↓90%	↓99%	↓45%	↓36%	Protected
LY294002	PI3K, CK2, EGFR, Wnt	10	N/A	↓45%	↓52%	↔	↓36%	↓42%	↓56%	None
Wortmannin	PI3K	1	N/A	↔	↑27%	↑70%	↑48%	↔	↔	ND
P110α siRNA	PI3K	N/A	N/A	↔	↔	↔	↑23%	↑23%	↔	ND
TBB	CK2	5	N/A	↔	↑25%	↑62%	↔	↔	↔	ND
β-catenin siRNA	Wnt	N/A	N/A	↔	↔	↔	↔	↔	↔	ND
AG1478	EGFR	5	no	↓41%	↔	↔	↔	↓52%	↓47%	Protected
NLRP3 siRNA	inflammasome	NA	N/A	↑47%	↔	↔	↔	↔	↔	ND
YVAD-CMK	caspase-1	10	no	↔	↔	↔	↔	↔	↔	ND
CLI-095	TLR4	10	yes	↓62%	↓92%	↓96%	↓98%	↓69%	↓81%	Protected
LPS 50 µg/ml	TLR4	50 µg/ml	yes	↓69%	↓65%	↓30	↓80%	↓88%	↓79%	Protected
ATF4 siRNA	EGFR, ER stress	N/A	N/A	↔	↔	↔	↔	↔	↔	None
CHOP siRNA	EGFR, ER stress	N/A	N/A	↔	↔	↓59%	↔	↓81%	↔	None
TOLLIP overexpression	IRAK4	N/A	N/A	↔	↔	↔	↔	↔	↔	ND
ST2825	MyD88 (TLRs)	10	N/A	↔	↓48%	↓43%	↔	↔	↔	ND
IRAK1/4 inhibitor	IRAK1/4	10	N/A	↔	↓58%	↔	↔	↔	↔	ND
Amlexanox1	IKKε/TBK	10	N/A	↑34%	↔	↑46%	↑35%	↑42%	↑49%	ND
Necrostatin	RIP1	10	N/A	↑38%	↑47%	↑42%	↑32%	↑43%	↑39%	ND
TRAF1 overexpression	TRIF	N/A	N/A	↔	↓63%	↓55%	↓47%	↓24%	↔	ND
BI-D1870	RSK1-4, PLK1	10	yes	↓63%	↓89%	↓75%	↔	↔	↔	Protected
SL0101	RSK1,2	10	N/A	↔	↔	↔	↔	↓21%	↔	Protected
SOCS2 overexpression	multiple	N/A	N/A	↓26%	↓48%	↓37%	↓34%	↓21%	↓18%	ND
SOCS3 overexpression	multiple	N/A	N/A	↔	↓38%	↓40%	↔	↔	↔	ND

This table summarizes all of the measured interactions mentioned in this study.

Definitions: N/A, not applicable, ↔ no statistically significant effect, ↓ down regulation, ↑ up regulation, ND, not determined.

Our *in vitro* and *in vivo* data strongly indicate that most of the signaling is occurring via the TLR4 receptor with some interconnection with EGFR-related pathways. This is in agreement with some of the previously published work (18, 43). However, our findings contradict other work which claims that 7KCh-induced inflammation is independent of Toll-like receptors [66].

The EGFR signaling is complex [67] and known to be involved in multiple diseases especially certain types of cancer [68]. The significant attenuation in the 7KCh-induced inflammatory response observed with the EGFR inhibitors LY294002 and AG1478 (Figs 4a and 7) supports the involvement of this receptor especially in regards to the ATF4/CHOP/GRP78/VEGF responses (Table 2, Fig. 7). The JAK/STAT pathway is one of the most important of the EGFR-related pathways which may also be involved in some of these responses (Fig. 19). Although we have not investigated this pathway directly the observed effects by SOCS2 and 3 overexpression indirectly implicate it since they are known to bind to JAK and STAT3 [58]-[60]. RSKs are also known to signal downstream of the EGFR Ras/Raf signaling pathway [69] (Fig. 19). However, inhibition of RSKs with BI-D1870 only affected VEGF, IL-1β and IL-6. Thus, it is unclear as to whether the reported ATF4 activation by RSKs [68] is occurring in our system. The evidence suggests different and yet unidentified kinases are involved. The inhibition observed by SA, BI-D1870 and SL0101 suggests that RSKs as well as other unidentified kinases are important in the interconnections between the EGFR and the TLR4 signaling. The lack of effect by the ERK1/2 inhibitor U0126 (Fig. 2b) suggests that the RSKs may be phosphorylated by a different upstream kinases. The potent effect of CLI-095 in in attenuating the immune response *in vitro* and *in vivo* (Fig. 9) as well as its protection from 7KCh-induced cell death (Fig. 11a) suggests that TLR4 is likely the primary response which can subsequently activates the EGFR-related responses. This activation may be due to 7KCh-induced release of TGFβ, although this has not been verified. A schematic showing the known and the speculative interactions are shown in figures 19 and 20.

Inhibition of the MyD88/TIRAP side of the receptor at MyD88 and IRAK1/4 had the most significant effect on IL-1β expression (Fig. 15). The MyD88 inhibition also had a modest effect on IL-6 and GRP78 suggesting they may be interacting with other unidentified kinases that signal downstream to NFκB (Fig. 20). This still suggests that most of the signaling was occurring through the TRIF/TRAM side of the receptor. Inhibition TRIF/TRAM side by targeting the RIP1 kinase and IKKε/TBK1 complex enhanced the 7KCh-induced inflammation (Fig. 16 a, b). However, overexpression of TRAF1 (a TRIF inhibitor) decreased the induction of IL-1β, IL-6, IL-8 and CHOP (Table 2, Fig. 16c). This supports the assumption that most of the cytokine signaling is occurring via TRIF/TRAM. The synergistic effect in the induction observed by RIP1 and the IKKε/TBK1 complex inhibition suggests these molecules are more involved in modulating the TLR4 response than in mediating it (at least in the case of 7KCh-induction). This also suggests that other downstream kinases are mediating the 7KCh-activation of the TLR4-TRIF/TRAM. The RSKs are likely a big part of the downstream signaling by TLR4 since their inhibition has significant effects on the inflammatory response *in vitro* and *in vivo* (Fig. 17). The RSK inhibitor BI-D1870 essentially ablated the angiogenesis response in our *in vivo* model (Fig. 17e). The differences observed between BI-D1870 and SL0101 are also interesting. SL0101 is not as potent as BI-D1870 but seems to only affect RSK1/2 [56]. SL0101 has no effect on reducing 7KCh-induced inflammation *in vitro* (Fig. 17b) and did not reduce angiogenesis in our *in vivo* model (data not shown), yet it provided significant protection from 7KCh-induced cell death. This would suggest that activation of RSK1/2 can induce the cell death pathway and that this pathway is independent of the inflammatory pathway. The effects of SL0101 are almost precisely opposite of the results observed with LY294002 (and MPK2 overexpression) which inhibits the inflammatory response (Fig. 4a) but does not protect from cell death (Fig. 11 e). However, since RSK1/2 are presumed to be upstream of NFκB, this would contradict the protection observed during the NFκB inhibition (Fig. 3). Without additional experimentation the only explanation we can offer is that the NFκB-related responses occur early (within 3 hr after 7KCh treatment) and the cell death requires 20 hr or more. This would give time for NFκB to induce the transcription of an RSK1/2 activator that can induce the cell death pathway.

SOCS are important immunoregulators that are known to interact with JAK/STAT as well as the MyD88/IRAK1/4 signaling [58], [59]. Overexpression of SOCS2 caused statistically significant suppression of all of the 7KCh-induced inflammatory markers (Fig. 18d). However, the ER stress markers were less significantly affected (Fig. 18d). Overexpression of SOCS3 also causes significant suppression of IL-1β and IL-6 (Fig. 18e). The known interactions by the SOCSs further support the involvement of these pathways. However, the multiple interactions by which these proteins perform their functions [58]–[60] make it difficult to pinpoint the exact molecules mediating the 7KCh-induced signaling without further experimentation.

The ER stress response caused by 7KCh is complex and involves practically all of the main ER stress components (Fig. 12). This response does not seem to follow the PI3K-calcium-UPR response. Instead it seems to be mediated by some unidentified kinases that are not RSKs (Fig. 17a,b) but may be somewhat affected by SOCS2 (Fig. 18d). This is supported by the fact that SA (a suspected kinase inhibitor) can attenuate and/or ablated all of the 7KCh-induced ER stress responses (Fig. 12) [19] SA seems to interact with 16 different kinases (Table 1) but aside from the RSKs the others have yet to be investigated.

In summary, we have used an *in vitro* and an *in vivo* experimental model to investigate 7KCh-induced inflammation. The interactions are complex but we can conclude that the majority of the signaling is through the TLR4 receptor which can interact and/or activate some of the EGFR-related pathways. Our data also suggests that the RSKs may be some of the downstream kinases that interconnect these two pathways.
